# Role of probiotic as adjuvant in treating various infections: a systematic review and meta-analysis

**DOI:** 10.1186/s12879-024-09259-3

**Published:** 2024-05-21

**Authors:** Erni Juwita Nelwan, Allerma Herdiman, Ayers Gilberth Ivano Kalaij, Richella Khansa Lauditta, Syarif Maulana Yusuf, Eva Suarthana

**Affiliations:** 1 Faculty of Medicine, Universitas Indonesia, DKI Jakarta, 10430 Indonesia; 2 Division of Tropical and Infectious Disease, Department of Internal Medicine, Faculty of Medicine, Universitas Indonesia, DKI Jakarta, 10430 Indonesia; 3 Infectious Disease and Immunology Research Center, Indonesia Medical and Education Research Institute (IMERI), Faculty of Medicine, Universitas Indonesia, DKI Jakarta, 10430 Indonesia; 4 Health Technology Assessment Unit (TAU) of the McGill University Health Center, Montreal, Canada

**Keywords:** *Helicobacter pylori*, Diarrheal infection, Urinary tract infection, Human immunodeficiency virus, Probiotics

## Abstract

**Background:**

Research on the advantages of probiotics has attracted increasing interest based on the number of publications, products, and public awareness of their benefits. This review evaluated the role of probiotics (single and multiple regimens) as an additional regimen to treat common infectious diseases, including *Helicobacter. pylori*, diarrheal infections, urinary tract infections (UTIs), upper respiratory tract infections (URTIs), and HIV infections.

**Methods:**

We searched randomized controlled trials from PubMed, Scopus, Embase, and Cochrane and identified 6,950 studies. Duplicates were removed, and titles and abstracts were filtered. Bias was evaluated using the Cochrane Risk of Bias Tool for Randomized Trials (ROB 1.0 and 2.0). The certainty of the evidence was evaluated using GRADE. Data were extracted and meta-analysis was performed using RevMan.

**Results:**

A total of 32 studies were included in this study (22 *H. pylori* studies, 2 diarrheal infection studies, 6 UTI studies, and 2 HIV infection studies). There was no study on URTI. Probiotics, in addition to primary treatment, could improve the eradication of *H. pylori* versus the control (RR: 1.09; 95% CI:1.04 − 1.13, *p* value = 0.001) and achieve a cure range of Nugent score in UTI patients (RR 1.38; 95% CI: 1.01 − 1.89, *p* value = 0.04). For eradicating *H. pylori* infection, subgroup analysis based on the therapy regimen showed that standard triple therapy was slightly superior compared to quadruple therapy in eradicating *H. pylori* (RR: 1.14 vs. 1.01, respectively). Single strain probiotics showed a similar effect to multiple strain probiotic regimens (both had an RR of 1.09). The effect estimates of the use of single strain probiotics as adjuvant therapy in eradicating H. pylori and the use of probiotics in UTI had a high certainty of evidence. Meta-analysis was not performed for infectious diarrheal because there were only two eligible studies with different probiotic supplementations and outcome parameters. Nonetheless, they showed that the diarrheal incidence was lower and complete remission of diarrheal was higher after the regimen of probiotics. Similarly, a meta-analysis was not performed for HIV infection because the two eligible studies used different designs and comparators with contradicting findings.

**Conclusion:**

This meta-analysis showed beneficial use of single strain probiotics as adjuvant therapy in eradicating H. pylori and the use of probiotics in UTI. Probiotic supplementation might not be beneficial for patients given a quadruple therapy. Single-strain and multi-strain probiotic regimens had similar effects in increasing the eradication rate of *H. pylori.* Our study also suggested that the benefits of probiotics as an additional regimen in infectious diarrheal and HIV infections remain unclear; more studies are needed to confirm the benefits.

**Supplementary Information:**

The online version contains supplementary material available at 10.1186/s12879-024-09259-3.

## Introductions

Until the twentieth century, the largest global burden of premature death and disability was mostly caused by infectious diseases [16]. Heretofore, vaccines, and curative treatments have become the ultimate approaches to preventing and treating infections. Although these approaches against infectious diseases are effective, other emerging pandemic infections remain a constant threat. For the past few years, probiotics have received much attention from studies demonstrating their ability to treat human diseases [32]. Probiotics are assumed to have a positive impact on human health by stimulating the immune system and inhibiting pathogens [61].

According to the Food and Agriculture Organization of the United Nations (FAO) and WHO, probiotics are consumable living organisms capable of inducing beneficial effects on human health [38]. Recent studies have demonstrated the ability of probiotics to boost human immunity, hence preventing the colonization of pathogens and reducing the number and severity of infections. Nevertheless, the underlying methods of probiotic mechanisms against infecting pathogens are largely unknown.

To date, studies have theorized that probiotics are involved in maintaining the balance and the stability of the gut microbiota by regulating the composition of the intestinal flora, maintaining the epithelial barrier, inhibiting pathogens from adhering to the intestinal surface, and modulating and properly maturing the immune system [59]. In the immune system, probiotics strengthen both innate and adaptive immune responses through bacterial-epithelial-immune cell crosstalk by acting as Toll-like receptors (TLRs) and modulating dendritic cells (DCs) [39].

Previous studies have proven probiotics' ability to reduce the risk of infectious diseases and the use of antibiotics as one of their broad functions [34]. For instance, the regimen of probiotics with antibiotics reduces the risk of AAD in adults by 37%, according to a study in Australia. In subgroup analyses, a high dose compared with a low dose of the same probiotic demonstrated positive protection [18]. Another study that included children, adults, and elderly individuals to assess probiotic effectiveness and safety in the prevention of acute URTIs showed that probiotic consumption is likely to reduce the number of participants diagnosed with URTIs, the incidence rate of URTIs, the mean duration of an episode of acute URTIs, and the number of participants who used prescribed antibiotics for acute URTIs [65]. The effect of probiotics in treating human immunodeficiency virus (HIV) infections benefits the CD4 count and may reduce immune activation and bacterial translocation thus reducing the acquisition or transmission of infections [5]. Furthermore, probiotics also improved the eradication rate and reduced side effects when added to the treatments designed to eradicate *H. pylori* [24].

The consumption of probiotics conceivably can improve immune function and prevent infectious diseases. However, more evidence is needed to investigate the effectiveness of probiotics as an additional regimen in treating infectious diseases. In this study, we analysed probiotic function as an adjuvant therapy in treating common infectious diseases including *H. pylori*, infectious diarrheal, urinary tract infections (UTIs), upper respiratory tract infection (URTI), and human immunodeficiency virus (HIV) infections.

## Methods

This systematic review and meta-analysis were conducted according to the Preferred Reporting Items for Systematic Review and Meta-Analysis (PRISMA) 2020 guidelines, which can be accessed through http://www.prismastatement.org/. The study protocol of this study was registered on the International Prospective Register of Systematic Reviews PROSPERO (CRD42022345021). No amendments to the protocol were needed.

### Information sources and search strategy

Four authors (AH, SMY, RKL, and AGIK) systematically searched the PubMed, Scopus, Embase, and Cochrane databases using the keywords (“probiotics” and “*H. pylori*” or “*Helicobacter pylori*”); (“probiotics” and “ID” or “Infectious-diarrhea”); (“probiotics” and “URTI” or “Upper Respiratory Tract Infection”); (“probiotics” and “UTI” or “Urinary Tract Infection”); and (“probiotics” and “HIV” or “Human Immunodeficiency Virus”) from January 2012 until 25th January 2024. The search was also conducted for unpublished trials through ClinicalTrials.gov. The reference lists of eligible articles were searched manually to identify additional literature. Supplementary Data 1 (a) displays a table of the source database and (b) table of the search strategy of every database, including detailed keywords used.

### Study eligibility criteria and determination of main outcome indicators

Following the literature search, studies were further screened using predetermined inclusion and exclusion criteria. All studies published in English in the last ten years assessing the role of probiotics in treating infectious diseases were included. The inclusion criteria used in this study were (1) RCT; (2) adults with infections defined as *H. pylori* infection, ID, UTIs, RTIs, or HIV infection, without a prior history of having the disease to adjust for confounding factors; (3) giving probiotics in addition to standard therapy, defined as triple or quadruple antibiotics or Proton Pump Inhibitor (PPI) for *H. pylori* infection; antiretroviral therapy (ART) for HIV; and antibiotic for ID, UTIs, URTIs, as their intervention; (4) placebo or conservative treatment only as their control; (5) cure or clinical improvement parameters as their outcome, defined as *H. pylori* eradication rates*,* achieved bristol stool scale for ID, Nugent score for UTI, improvement of CD4^+^ for HIV. The diseases chosen were the five common infectious diseases in Indonesia. This study excluded (1) cadaveric or animal studies; (2) studies with no follow–up; (3) studies in infants, children, or young adults; (4) studies with mixed subject ages; and (5) probiotic prevention studies. The outcomes of this study are defined further in Table 1.

**Table 1:** Operational definition of cure in every disease included

DEFINITION OF CURE IN EVERY DISEASE	
*H. pylori*	The definition of eradication rates in *H. pylori* infections is the percentage of patients who are cured of *H. pylori* infection or have a negative Urea Breath Test (UBT) result per total patients who received treatments [3]
Infectious Diarrhea	Type 3,4,5, are considered the normal stool forms that indicate the patients are cured from diarrhea [4]
URTI	Improvement of infection marker compared to baseline [60]
UTI	Nugent score 0–3 considered as Bacterial Vaginosis (BV) negative Nugent score 4–6: intermediate microbiota Nugent score 7- 10: BV Positive [23]
HIV	An increase in CD4^+^ cells indicates immunological cure in HIV patients [1]

### Study selection

**Fig. 1:**
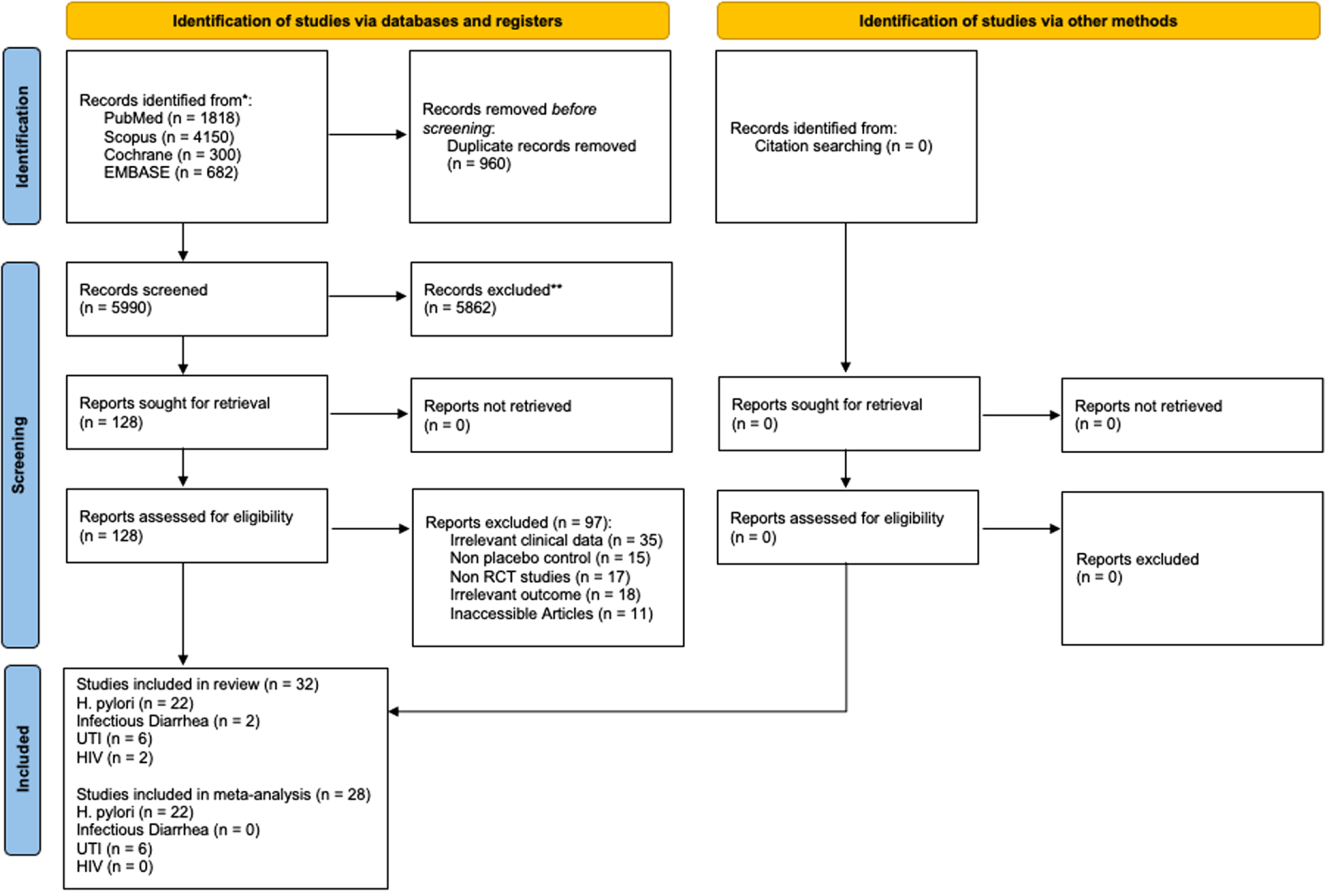
Diagram Flow of Searching Strategies

Duplicates were removed prior to title and abstract screening using EndNote X9 Software and Mendeley Desktop Software. Furthermore, title and abstract screening of the included studies was performed according to study eligibility criteria by four independent reviewers (AH, SMY, AGIK, and RKL). Disagreements were then discussed further until a consensus was reached. A detailed planned literature search procedure is illustrated in Fig. 1.

#### Data extraction

Four reviewers (AH, SMY, AGIK, and RKL) independently extracted data, which were then discussed to reach a consensus. Data extracted included: author and publication year; study design; study location; subject characteristics; follow-up durations; interventions (including the types of probiotics); and outcomes per disease, which were stated according to the disease cure or clinical improvement parameters. Studies were grouped according to the diseases assessed.

#### Quality assessment

The included studies were also assessed in terms of their quality using the Cochrane Risk of Bias Tool for Randomized Trials (RoB 1.0 and ROB 2.0) (Supplementary Data 2). Results of RoB 1.0 and RoB 2.0 were then compared to ensure the quality of the studies assessed. The quality assessment was performed by four reviewers (AH, SMY, RKL, and AGIK) with each other blinded to each other's scoring and then discussed until consensus was reached. A funnel plot was also used to determine publication bias if the study included for each group was more than 10, as recommended by the Cochrane Handbook. GRADE Assessment (Supplementary Data 3) was also done to assess the quality of evidence among included studies. A completed PRISMA checklist is displayed in Supplementary Data 4.

#### Data synthesis

We analysed the data using Review Manager software (RevMan v5.4). We calculated the pooled estimates as the risk ratios (RRs), both with the corresponding 95% confidence intervals (CIs). Statistical heterogeneity among studies was evaluated by I^2^ with values of 0–40%, suggesting a low heterogeneity. We utilized fixed effect models for the meta-analysis of trials with low heterogeneity and random effect models for trials with high heterogeneity. Subgroup analysis was performed for therapy (triple vs. quadruple) and probiotic regimens (single vs. multiple strains) based on risk ratios. Furthermore, sensitivity analyses were performed using Duval and Tweedie’s trim-and-fill analysis.

## Results

### Search results and study selection

The initial literature search yielded a total of 6,950 studies, detailing 1,818 from PubMed, 4,150 from Scopus, 300 from Cochrane, and 682 from EMBASE. After the deletion of duplicates, titles, and abstracts were screened, and a total of 128 studies were obtained to be evaluated for eligibility evaluation. Due to irrelevant clinical data, 35 studies were subsequently excluded. Furthermore, 15 were non-placebo studies, 17 were non-RCT studies, 18 were studies with irrelevant results, and full texts were not available for 11 studies. All rejected articles and the reason for rejection are provided in Supplementary Data 4. As a result, we reviewed 32 studies, detailing 22 *H. pylori* studies, 2 diarrheal infection studies, 6 UTI studies, and 2 HIV studies (Fig. 1).

### Study characteristics and findings

Overall, this review included a total of 6,509 patients (Table 2), detailing 4721 patients from 22 *H. pylori* studies, 1194 patients from 2 ID studies, 552 patients from 6 UTI studies, and 42 patients from 2 HIV studies. The study locations were spread across Asia, America, and Europe. The outcomes of the study were defined as the cures described in our methods. The study characteristics and findings of the included studies are displayed in Table 2.

**Table 2:** Table of study characteristics

**Author and Publication Year**	**Study Design**		**Study Location**	**Subject Characteristics**	**Intervention**	**Follow-up Duration**	**Outcomes**	
	**Study Phase**	**Blinding**					**Parameter used**	**Cure Results**
a. ***H. pylori* infections** with single probiotic regimen (*Bifidobacterium*)								
Cekin et al., 2017 [6]	NR	Single- blind	Antalya, Turkey	159 patients were diagnosed with *H. pylori* via endoscopic gastric biopsies **Group I (ERA + Probiotic)** as I: - n: 53 - mean ± SD age: 47.7 ± 14.0 - 45.3% were males **Group II (ERA + Placebo)** as C: - n: 52 - mean ± SD age: 46.4 ± 13.4 - 51.9% were males Group III (ERA only): - n: 54 - mean ± SD age: 46.3 ± 11.9 - 44.4% were males	All patients received 2 weeks of STT with amoxicillin 1000 mg + PPI (Pantoprazole 40 mg) in the first week and then metronidazole 500 mg + clarithromycin 500 mg + PPI 40 mg in the second week) At the same time, the patients were divided into three arms: **Group I (ERA + Probiotic)**: Patients received a probiotic supplement with Maflor® (7 × 10^9^ CFU *B. animalis* subsp. lactis B94; 1 capsule/day) **Group II (ERA + Placebo)**: Patients received placebo treatment (1 capsule/day) Group III (ERA only): Patients received no additional treatments	4 weeks after the end of therapy	Evaluation of the *H. pylori* status was repeated via a ^14^C UBT	I (Group I): 86.8% *P* (Group II): 69.2% *p*-value: 0.003 (S)
Chitapanarux et al., 2015 [9]	NR	Double blind	Chiang Mai, Thailand	63 patients with dyspeptic complaints Intervention group: - mean ± SD age: 52.6 ± 11.3 - 45.2% were males Placebo group: - mean ± SD age: 49.4 ± 13.3 - 43.7% were males	1 week-standard triple therapy (esomeprazole 40 mg, amoxicillin 1000 mg, clarithromycin 500 mg) twice a day + 4 weeks-probiotics Combif AR® (*B. longum* BB536; 2 capsules/twice a day or placebo; 2 capsules/twice a day, or as a placebo	4 weeks after the end of the eradication therapy	Evaluation of the *H. pylori* status was repeated via a ^13^C UBT	I: 93.33% P: 73.33% *p*-value: 0.040 (S)
Dajani et al., 2013 [11]	NR	Single- blind	Dubai, Uni Emirates Arab	206 patients with upper gastrointestinal symptoms **Group A** (as Control/placebo): - n: 106 - mean age: 37.2 - 48.1% were males **Group B** (as Intervention): - n: 100 - mean age: 37.3 - 51% were males Group C (): - n: 100 - mean age: 37.3 - 51% were males Group D (): - n: 76 - mean age: 38.3 - 50% were males	All patients received 2 weeks of STT with PPI, amoxicillin 1000 mg, clarithromycin 500 mg, or metronidazole 400 mg) twice a day Then, the patients were divided into four arms: **Group A (STT only or as placebo group)**: Patient received no additional treatments **Group B (as Intervention group**): Patients received a probiotic supplement with *B. infantis* 2036 at 3 × 10^9^ CFU (twice daily) for 10 days Group C: Patients were planned for a lead-in period of 2 weeks with probiotic *B. infantis* 2036 at 3 × 10^9^ CFU (twice daily) alone, then followed by triple therapy combined with *B. infantis* as an adjuvant (same as in group B) for the subsequent 10 days Group D: Patients treated with STT regimen together with *B. infantis* 2036 at 3 × 10^9^ CFU (twice daily) for 10 days	6–8 weeks after the end of the eradication therapy	Evaluation of the *H. pylori* status was repeated via a ^14^C UBT	I: 83% C: 68.9% *p*-value: 0.001 (S)
b. ***H. pylori* infections** with single probiotic regimen (*Lactobacillus*)								
Deguchi et al., 2012 [12]	NR	Single- blind	Kanagawa, Japan	229 patients diagnosed with an *H. pylori* infection Intervention group: - mean age: 55.9 - 66.1% were males Placebo group: - mean age: 57.8 - 57.9% were males	1 week: STT (rabeprazole 10 mg, amoxicillin 570 mg, clarithromycin 200) + 4 weeks-probiotics (*L. gasseri* OLL2716 yogurt; 112 g) twice daily (3 weeks pretreatment followed by 1 week during eradication therapy), or as a placebo	8 weeks after the end of the eradication therapy	Evaluation of the *H. pylori* status was repeated via a ^13^C UBT	I: 85.6% P: 74.5% *p*-value: 0.041 (S)
Emara et al., 2014 [15]	NR	Double-blind	Zagagig, Egypt	213 patients with dyspeptic symptoms Group A (as Intervention): - mean age: 33.2 ± 13.9 -37.1% were males Group B (as Placebo): - mean age: 36.9 ± 11.1 - 31.4% were males	2 weeks-STT (omeprazole 20 mg, amoxicillin 1000 mg, clarithromycin 500 mg) twice a day + 4 weeks-probiotics 1 capsule of *L. reuteri* DSM 17938, *L. reuteri* ATCC PTA 6475, 1 × 10^8^ CFU twice a day (2 weeks probiotics alone, then followed by 2 weeks during eradication therapy), or as a placebo	4 weeks after the end of the eradication therapy	Evaluation of the *H. pylori* status was used GSRS score	I: 74.3% P: 65.7% *p*-value: 0.603 (NS)
Francavilla et al., 2014 [17]	NR	Double-blind	Bari, Italy	478 consecutive patients with dyspepsia Intervention group: - mean age: 49.0 - 36% were males Placebo group: - mean age: 44.0 - 42% were males	1 weeks-STT (clarithromycin, amoxicillin, and PPI) + probiotics (*L. reuteri* DSM 17938 and *L. reuteri* ATCC 6475, dose 2 × 10^8^ CFU) 1 capsule daily, or as a placebo	4 weeks after the end of the eradication therapy	Evaluation of the *H. pylori* status was repeated via a ^13^C UBT	I: 76.7% P: 67.4% *p*-value: > 0.050 (NS)
Ismail et al., 2023 [28]	NS	Double Blind	Kuala Lumpur, Malaysia	90 patients diagnosed with an *H. pylori* infection Probiotic group: - median age: 49 (37.5–68.8) - 52.6% were males	2 weeks STT (amoxicillin clarithromycin, esomeprazole) + 4 weeks- 1 capsule (200 mg) probiotic or placebo once daily	8 weeks from baseline of eradication	Evaluation of the *H. pylori* status was repeated via a ^14^C UBT	I: 93.2% P: 68.9% *p*-value: < 0.001 (S)
c. ***H. pylori* infections** with single probiotic regimen (*Saccharomyces*)								
Seddik et al., 2019 [48]	NR	Single-blind	Rabat, Morocco	199 patients with *H. pylori* infection confirmed by endoscopic gastric biopsy Intervention group: - mean age ± SD: 43.2 ± 13.2 - 46.7% were males Placebo group: - mean age ± SD: 46.3 ± 13.8 - 53.2% were males	10 days: STT (omeprazole 20 mg, amoxicillin 1 g) twice daily for 5 days, then followed by twice daily 5 days of triple therapy (omeprazole 20 mg, clarithromycin 500 mg, metronidazole 500 mg) + 10 days of probiotics (*S. boulardii* 250 mg), or as a placebo	4 weeks after the end of the eradication therapy	Evaluation of the *H. pylori* status was repeated via a ^13^C UBT	I: 87.5% P: 78.9% *p*-value: 0.040 (S)
Zhao et al., 2021 [66]	NR	Double-blind	Hubei, China(Torres et al., 2014)	360 patients with *H. pylori* infection Group B (as Intervention): - mean age ± SD: 45.3 ± 11.5 - 50.9% were males Group A (as Placebo): - mean age ± SD: 46.7 ± 12.8 - 54.3% were males	2 weeks- SQT (esomeprazole 20 mg, amoxicillin 1 g, clarithromycin 500 mg, bismuth potassium citrate) + 2 weeks- probiotics (*S. boulardii* 500 mg), or as a placebo	4 weeks after the end of the eradication therapy	Evaluation of the *H. pylori* status was repeated via a ^13^C/^14^C UBT	I: 94.2% P: 89.7% *p*-value: 0.146 (NS)
Zojaji et al., 2013 [67]	NR	Single- blind	Tehran, Iran	160 patients with *H. pylori* infection All group: - mean age ± SD: 47.1 ± 11.4 - 41.3% were males	2 weeks-STT (amoxicillin 1000 mg, clarithromycin 500 mg, omeprazole, 30 mg) + 2 weeks-probiotics (*S. boulardii* 250 mg), or as a placebo	8 weeks after the end of the eradication therapy	Evaluation of the *H. pylori* status was repeated via UBT	I: 87.5% P: 81.2% *p*-value: 0.350 (NS)
d. ***H. pylori* infections** with single probiotic regimen (*Clostridium*)								
Chen et al., 2018 [8]	NR	Single- blind	Zhejiang, China	Of the 70 patients enrolled, *H. pylori* positive gastritis was diagnosed by esophago-gastro-duodenoscopy Group A (as Placebo): - mean age ± SD: 46.7 ± 12.8 - 54.3% were males Group B (as Intervention): - mean age ± SD: 45.3 ± 11.5 - 50.9% were males	Group A (as Placebo): 2 weeks-SQT (pantoprazole 40 mg, amoxicillin 1000 mg, furazolidone 100 mg, colloidal bismuth pectin 0.4 g) twice a day Group B (as Intervention): 2 weeks-SQT same with group A + probiotics (*Clostridium butyricum*, 40 mg, 3 times/day)	8 weeks after the end of the eradication therapy	Evaluation of the *H. pylori* status was repeated via ^13^C UBT	I: 96.9% P: 96.8% *p*-value: 1.000 (NS)
e. ***H. pylori* infections** with multiple probiotic regimens								
Dore et al., 2019 [14]	NR	Single- blind	Sassari, Italy	99 patients diagnosed with dyspeptic symptoms and found positive for *H pylori* infection were studied Group I (as Intervention): - mean age ± SD: 54.1 ± 14 - 32.6% were males Group II (as Placebo): - mean age ± SD: 52.2 ± 14 - 54.3% were males	Group I (with Probiotic): 10 days-SQT (pantoprazole 20 mg, tetracycline 500 mg, metronidazole 500 mg, PPI) twice a day + 4 weeks-probiotic supplement with Gastrus® 1 capsule (2 × 10^8^ CFU of *L. reuteri* DSM 17 938 and 2 × 10^8^ CFU of *L. reuteri* ATCC PTA 6475) once daily Group II (Placebo): 10 days-pantoprazole 20 mg and the same doses of antibiotics administered as tetracycline 250 mg and metronidazole 250 mg plus Pylera® 2 capsules twice a day	4 weeks after the end of the eradication therapy	Rates of eradication at 4 weeks after the end of therapy	I: 84.8% P: 95.7% *p*-value: 0.255 (NS)
Grgov et al., 2016 [21]	NR	Single- blind	Leskovac, Serbia	167 patients with endoscopic and histological findings of chronic gastritis Group I (as Placebo): - mean age ± SD: 56.2 ± 14.8 - 35.1% were males Group II (as Intervention): - mean age ± SD: 56.3 ± 14.8 - 46.7% were males -	Group I (Placebo): 5 weeks- in the first week were treated with STT (lansoprazole 2 × 30 mg, amoxicillin 2 × 1000 mg, clarithromycin 2 × 500) after the 7th day of the therapy, lansoprazole was continued in dose of 30 mg for 4 weeks Group II (with probiotic): were treated same STT as well as the patients in group 1 with an additional 1 capsule of probiotics containing *S. boulardii, Lactobacillus acidophilus* rosell-52*, Lactobacillus rhamnosus* rosell-11*,* and *B. longum* rosell-175, total 5 × 10^9^ CFU; once a day during lunch	8 weeks after the end of the eradication therapy	Evaluation of the *H. pylori* status was repeated via UBT	I: 93.3% P: 81.8% *p*-value: 0.05 (NS)
Haghdoost et al., 2017 [22]	NR	Single- blind	Tabriz, Iran	176 patients with dyspeptic symptoms Intervention group: - mean age ± SD: 54.1 ± 14 - 32.6% were males Placebo group: - mean age ± SD: 52.2 ± 14 - 54.3% were males	10 days-STT (pantoprazole 40 mg, amoxicillin 100 mg, clarithromycin 500 mg) + 4 weeks after therapy-probiotics supplement of Prodigest® 500 mg contains *Lactobacillus* and *Bifidobacterium* with a total of 15 × 10^8^ CFU/capsule, or as a placebo	4 weeks after the end of the eradication therapy	Evaluation of the *H. pylori* status was repeated via Toyo *H. pylori* antigen test stool	I: 78.4% P: 64.8% *p*-value: 0.033 (S)
Hauser et al., 2015 [24]	NR	Double-blind	Rijeka, Croatia	804 subjects with confirmed *H. pylori* infection All group: - mean age ± SD: 28.3 ± 5.8 - 53.9% were males	2 weeks-STT (omeprazole 2 × 20 mg, clarithromycin 2 × 500 mg, amoxicillin 2 × 1000 mg) + 2 weeks-probiotics supplement of Normia® contains *L. rhamnosus GG* (LGG®) and *Bifidobacterium* (BB-12®) with total 1 × 10^8^ until 1 × 10^10^, twice a day, or as a placebo	6 weeks after the end of the eradication therapy	Evaluation of the *H. pylori* status was repeated via UBT	I: 87.38% P: 72.55% *p*-value: 0.001 (S)
McNicholl et al., 2018 [41]	NR	Double-blind	Madrid, Spain	209 patients with *H. pylori* infection Intervention group: - mean age ± SD: 47 ± 13 - 40% were males Placebo group: - mean age ± SD: 45 ± 13 - 35% were males	10 days-STT (PPI at standard doses (e.g., omeprazole 20 mg), clarithromycin 500 mg, and amoxicillin 1 g) twice daily + 10 days-1 capsule probiotic formula combining 2 bacterial strain 1 × 10^9^ CFU for each strain of *Lactobacillus plantarum,* CETC7879 and *Pediococcus acidilactici* CETC7880), or as a placebo	6 weeks after the end of the eradication therapy	Evaluation of the *H. pylori* status was repeated via ^13^C-UBT	I: 97% P: 95.2% *p*-value: 0.721 (NS)
Rodriguez et al., 2013 [44]	NR	Double-blind	Sao Paulo, Brazil	107 patients with peptic ulcer or functional dyspepsia Intervention group: - mean age ± SD: NR - 38.2% were males Placebo group: - mean age ± SD: NR - 36.5% were males	7 days-STT (30 mg of lansoprazole, 500 mg tetracycline, 200 mg furazolidone (twice a day) + 4 weeks-probiotics formula combining 4 bacterial strain 1.25 × 10^9^ CFUs for each strain of *L. acidophillus, L. rhamnosus, Bifidobacterium bifidum,* and *Streptococcus faecium,* twice a day, or as a placebo (containing acidified milk powder was also provided at the same amount and with the same instructions)	4 weeks after the end of the eradication therapy	Evaluation of the *H. pylori* status was repeated via ^13^C-UBT	I: 89.8% P: 85.1% *p*-value: 0.490 (NS)
Shavaki et al., 2013 [51]	NR	Triple- blind	Isfahan, Iran	170 patients with peptic ulcer disease and confirmed *H. pylori* infection Intervention group: - mean age ± SD: 42.3 ± 13.3 - 54.4% were males Placebo group: - mean age ± SD: 42.2 ± 13.2 - 66,6% were males	2 weeks-SQT (20 mg omeprazole, 240 mg bismuth subcitrate, 1000 amoxicillin, 500 clarithromycin) + 2 weeks-probiotics formula combining seven bacterial strains with total count 1 × 10^8^ CFU/capsule (*Lactobacillus: L. casei, L. rhamnosus, L. acidophilus,* and *L. bulgaricus*; *Bifidobacterium: B. breve, B. longum; Streptococcus thermophile*), or as a placebo	4 weeks after the end of the eradication therapy	Evaluation of the *H. pylori* status was repeated via ^13^C-UBT	I: 76.6% P: 81.1% *p*-value: 0.292 (NS)
Srinarong et al., 2014 [54]	NR	Single- blind	Bangkok, Thailand	100 patients with *H. pylori* infection All group: - mean age: 50.5 - 28% were males	STT consisted of lansoprazole 30 mg (twice daily), amoxicillin 1 g (twice daily) and clarithromycin 1 g (once daily), bismuth subsalicylate 1.048 mg (twice daily) Probiotic yogurt composed of *Bifidobacterium lactis, L. acidophillus, and Lactobacillus paracasei* (≥ 10^9^ CFU/serve or as a placebo (conventional yogurt without probiotics) Then, the patients were divided into four arms: Group I: 7-day STT + probiotic Group II: 14-day STT + probiotic Group III: 7-day STT + placebo Group IV: 14-day STT + placebo	2 weeks after the end of the eradication therapy	Evaluation of the *H. pylori* status was repeated via rapid urea test	I_7-day probiotic (Group 1)_: 100% P_7-day placebo (Group III)_: 81.1% *p*-value: (NS) I_14-day probiotic (Group II)_: 100% P_14-day placebo (Group IV)_: 96% *p*-value: (NS)
Tang et al., 2021 [56]	NR	Single- blind	Chongqing, China	162 patients with *H. pylori* infection Intervention group: - mean age ± SD: 43.3 ± 11.3 - 71.4% were males Placebo group: - mean age ± SD: 45.3 ± 10.9 - 59.5% were males	2 weeks of SQT (esomeprazole 20 mg, amoxicillin 1000 mg, furazolidone 100 mg, bismuth potassium citrate 220 mg) twice daily + 4 weeks-probiotics supplement with Medilac-S contains *Enterococcus faecium* 4.5 × 10^8^ and *Bacillus subtilis* 5.0 × 10^7^, 3 times a day, or as a placebo (maltodextrin)	6 weeks after the end of the eradication therapy	Evaluation of the *H. pylori* status was repeated via ^13^C-UBT	I: 89.33% P: 84.72% *p*-value: 0.226 (NS)
Tongtawe et al., 2015 [57]	NR	Single- blind	Bangkok, Thailand	200 patients with *H. pylori* associated gastritis Intervention group: - mean age: 47.5 - 42.8% were males Placebo group: - mean age: 45.2 - 30.2% were males	1 week-STT (esomeprazole 20 mg, clarithromycin 500 mg, metronidazole 400 mg) + 1 week of pretreatment with probiotic containing *L. delbrueckii,* subsp. *bulgaricus*, and *S. thermophilus*), or as a placebo	4 weeks after the end of the eradication therapy	Evaluation of the *H. pylori* status was repeated via rapid urea test	I: 90.8% P: 84.3% *p*-value: 0.040 (S)
Tongtawe et al., 2015 [58]	NR	Single- blind	Bangkok, Thailand	300 patients diagnosed with *H. pylori* associated gastritis **Group I (Placebo)**: - n: 98 - mean age: 46.2 - 49% were males **Group II (with Probiotic before STT)**: - n: 97 - mean age: 55.9 - 49% were males Group III (with Probiotic before and after STT): - n: 100 mean age: 51%	**Group 1 (Placebo):** 1 week- STT (esomeprazole 20 mg, clarithromycin 500 mg, metronidazole 400 mg) + placebo **Group II (with Probiotic before STT):** 1 week-pretreatment with probiotics containing *Lactobacillus delbrueckii* subsp. *bulgaricus* and *Streptococcus thermophilus*) Group III (with Probiotic before and after STT): 1 week- pretreatment probiotic before tailored triple therapy then followed by 1 week of the same probiotic after treatment	4 weeks after the end of the eradication therapy	Evaluation of the *H. pylori* status was repeated via rapid urea test	I: 74.5% P: 77.3% *p*-value: 0.010 (S)
f. **Urinary Tract Infection**								
Cohen et al., 2020 [10]	IIb	Randomized Double-blind	United States of America	228 participants with BV, diagnosed by Nugent Score 4–7 Intervention group: - mean age ± SD: 30.7 ± 6.8 - 100% were females Placebo group: - mean age ± SD: 31.4 ± 7.1 - 100% were females	11 weeks-Metronidazole in combination with Lactin V at 2 × 10^9^ CFU contains *Lactobacillus crispatus* CTV-05, twice weekly or as a placebo The placebo formulation contained the same inactive ingredients as Lactin-V, without *L. crispastus* CTV-05	12 weeks after the probiotic treatment	The parameter used is Gram’s staining of the vaginal smear was used to determine the Nugent score; normal (0 to 3), intermediate (4 to 6), or indicative of bacterial vaginosis (7 to 10)	I: 57% P: 39% *p*-value: 0.010 (S)
Laue et al., 2017 [33]	II	Randomiezed double blind	Bad Segeber, Germany	48 participants with BV diagnosed by Nugent score Intervention group: - mean age ± SD: 32.6 ± 11.2 - 100% were females Placebo group: - mean age ± SD: 39.0 ± 12.3 - 100% were females	4 weeks-Metronidazole with Verum (yogurt contains *L. crispatus, L. gasseri, L. rhamnosus,* and *L. jensenii* with a total 1 × 10^7^ CFU/mL)*,* 2 yogurt drinks daily or as a placebo The placebo treatment consisted of daily 2 × 125 g chemically (with H_3_PO_4_) acidified milk without bacterial strains	4 weeks after the probiotic treatment	The parameter used is Nugent score 0–3	I: 81.3% P: 64.7% *p*-value: 0.438 (NS)
Sgibnev et al., 2019 [50]	II	Randomized Double-blind	Orenburg, Russia	86 of the participants with BV were diagnosed by Amsel’s Criteria Intervention group: - mean age ± SD: 25.3 ± 2.4 - 100% were females Placebo group: - mean age ± SD: 23.6 ± 2.1 100% were females	2 weeks-Metronidazole (2 × 500 mg) + 1 capsule of probiotic Gynophilus® vaginally (*Lactobacillus casei var. rhamnosus*) twice in a day, or as a Placebo	15 days after the probiotic treatment	The parameter used is Nugent score 0–3	I: 88.6% P: 42.9% *p* value’: < 0.001 (S)
Happel et al., 2020 [23]	II	Randomized single-blind	Cape town, south africa	43 of the participants were confirmed to have BV by Nugent’s criteria Intervention group: - mean age: 22 - 100% were females Placebo group: - mean age: 23 - 100% were females	5 days-topical metronidazole once a day with a 15 days-treatment of probiotic (*L. acidophilus, L. rhamnosus* GG, *B. bifidum, and B. longum* ≥ 2 × 10^9^ CFU) or as a placebo	20 weeks after the probiotic treatment	The parameter used is Nugent score 0–3	I: 33.8% P: 63.6% *p*-value: 0.109 (NS)
Russo et al, 2019 [47]	II	Randomized Double-blind	Romania	48 of the participants were confirmed to have BV by Nugent’s criteria Intervention group: - mean age ± SD: 35.4 ± 9.2 - 100% were females Placebo group: - mean age ± SD: 36.7 ± 7.7 - 100% were females	1 weeks-metronidazole oral twice daily with Verum (ingredients *L. acidophilus* LMG S29159 and *L. rhamnosus* ATCC SD5675) or placebo, 2 capsules/day for 5 days followed by 1 capsule/day for 10 days The placebo was an identical capsule containing the inactive ingredient maltodextrin (100 mg)	24 weeks after the probiotic treatment	The parameter used is Nugent score 0–3	I: 83.3% P: 37.5% *p*-value: < 0.010 (S)
Zhang et al., 2021 [64]	II	Randomized, single-center prospective parallel group	Peking, China	99 participants were confirmed BV by Nugent’s criteria Intervention group: - mean age ± SD: 34.2 ± 7.0 - 100% were females Placebo group: - mean age ± SD: 33.3 ± 7.5 - 100% were females	7 days-metronidazole suppositories with probiotics (*Lacticaseibacillus rhamnosus* GR-1 and *Limosilactobacillus reuteri* RC-14) drink or placebo The placebo was received metronidazole vaginal suppositories only	12 weeks after the probiotic treatment	The parameter used is Nugent score 0–3	I: 57.69% P: 59.57% *p*-value: 0.040 (S)
g. **Human Immunodeficiency Virus (HIV) infection**								
Hemsworth et al., 2015 [25]	Randomized, three-period, crossover controlled trial	Double-blinded	Ontario- Canada	25 patients stable HAART therapy All group: - mean age ± SD: 47.9 ± 9.3 - 75% were males	Antiretroviral (ART) with 3 treatment sequences: 1. Type A contained micronutrients 175 g (vit. A, vit E, Niacinamide, vit. B1, vit. B12, vit. B6, vit. C, iron, selenium, zinc, DHA, EPA) and *L. rhamnosus* CAN-1 (min 10^9^ CFU/mL) 2. Type B contained only micronutrients 3. **Type C: contained only *L. rhamnosus* CAN-1 (10**^**9**^** CFU/ML)** The period of intake for each of the types was 30 days with a 14-day wash-out period between the intervention types	Assessment of CD4 cell count was obtained on days 0 and 30	The parameter used is CD4^+^ cell increasing	CD4^+^ cell count increased on average (Mean Δ) by Type A: 19.2 ± 142 cells/uL (*p*-value: 0.543, NS) Baseline: 619 ± 316 Follow-up: 638 ± 384 Type B: 40.5 ± 221 cells/uL (p-value: 0.411, NS) Baseline: 569 ± 351 Follow-up: 637 ± 357 Type C: -6.6 ± 154 cells/uL (*p*-value: 0.845, NS) **Baseline: 654 ± 368** **Follow-up: 639 ± 357**
Yang et al., 2014 [62]	NR	Double-blind	Los Angeles, California	17 patients (10 probiotic, 7 placebo) with chronic HIV-1 infection Intervention group: - mean age: 50.4 - 100% were males Placebo group: - Mean age: 48.4 - 86% were males	Antiretroviral (ART) + 12 weeks received a daily/capsule probiotics (GanedinBC®) 2 × 10^9^ CFU of *Bacillus coagulans* GBI-30 or placebo	Assessment of CD4^+^ cell count was obtained at days 0 and 90	The parameter used is CD4^+^ cell increasing	**Intervention:** CD4^+^ cell count at Baseline: 485 ± 152 Follow-up: 508 ± 150 **Placebo:** CD4^+^ cell count at Baseline: 432 ± 155 Follow-up: 486 ± 229

### Risk of bias and certainty of evidence

Upon RoB 1.0 analysis, one study had a moderate risk of bias (Happel) and five studies (Grgov, Srinarong, Tang, Tongtawee 2015a, and Tongtawee 2015b) had a high risk of bias. Similarly, RoB 2.0 analysis showed that only one study had a moderate risk (Dajani) and only two studies had a high risk of bias (Grgov, Srinarong). Details of the bias of the studies are presented in Supplementary Data 2. GRADE Assessment (Supplementary Data 3) indicated that the effect estimates of the use of single strain probiotics as adjuvant therapy in eradicating H. pylori and the use of probiotics in UTI had a high certainty of evidence. The effect estimates in other subgroups: the use of probiotics as adjuvant to standard triple or quadruple therapy as well as the use of multiple strain probiotics as adjuvant therapy in eradicating H. pylori had a moderate certainty of evidence.

#### Probiotic and infectious diarrhea

A meta-analysis was not performed for infectious diarrhea because the two eligible studies used different probiotic supplementations and outcome parameters. Among acute diarrhea patients, Greuter et al. found that the diarrheal incidence after a regimen of a probiotic (*E. faecium*) three times a day for a week was lower (8.6%) than that after a regimen of a placebo (16.2%) (*p*-value < 0.001) [20]. Meity et al. showed that by giving probiotics (*B. coagulans*) with the same time and duration of regimen (three times a day for a week), the complete remission of diarrhea was 100% on day 5 of the probiotic regimen, while in the placebo group, it was only 26.7% (*p*-value < 0.001) [37].

#### Probiotic and HIV

A meta-analysis was not performed for HIV infection because the two eligible studies used different designs and comparators. Hemsworth et al., used a crossover design to evaluate micronutrients and probiotics (A), micronutrients alone (B), and probiotics alone (C). The highest mean increase in CD4 was obtained with micronutrients alone (41 cells/µL, SD 221). After a washout period and given a probiotic regimen alone, the mean CD4 level declined (-7 cells/µL, SD 154). Yang et al. performed a two-arm RCT with more promising results [62]. They found that the percentage of blood CD4( +) T cells in the probiotics group was higher than that in the placebo group (+ 2.8% versus -1.8%, *p* = 0.018).

### Meta-analysis: *probiotic and helicobacter pylori infection*

**Fig. 2:**
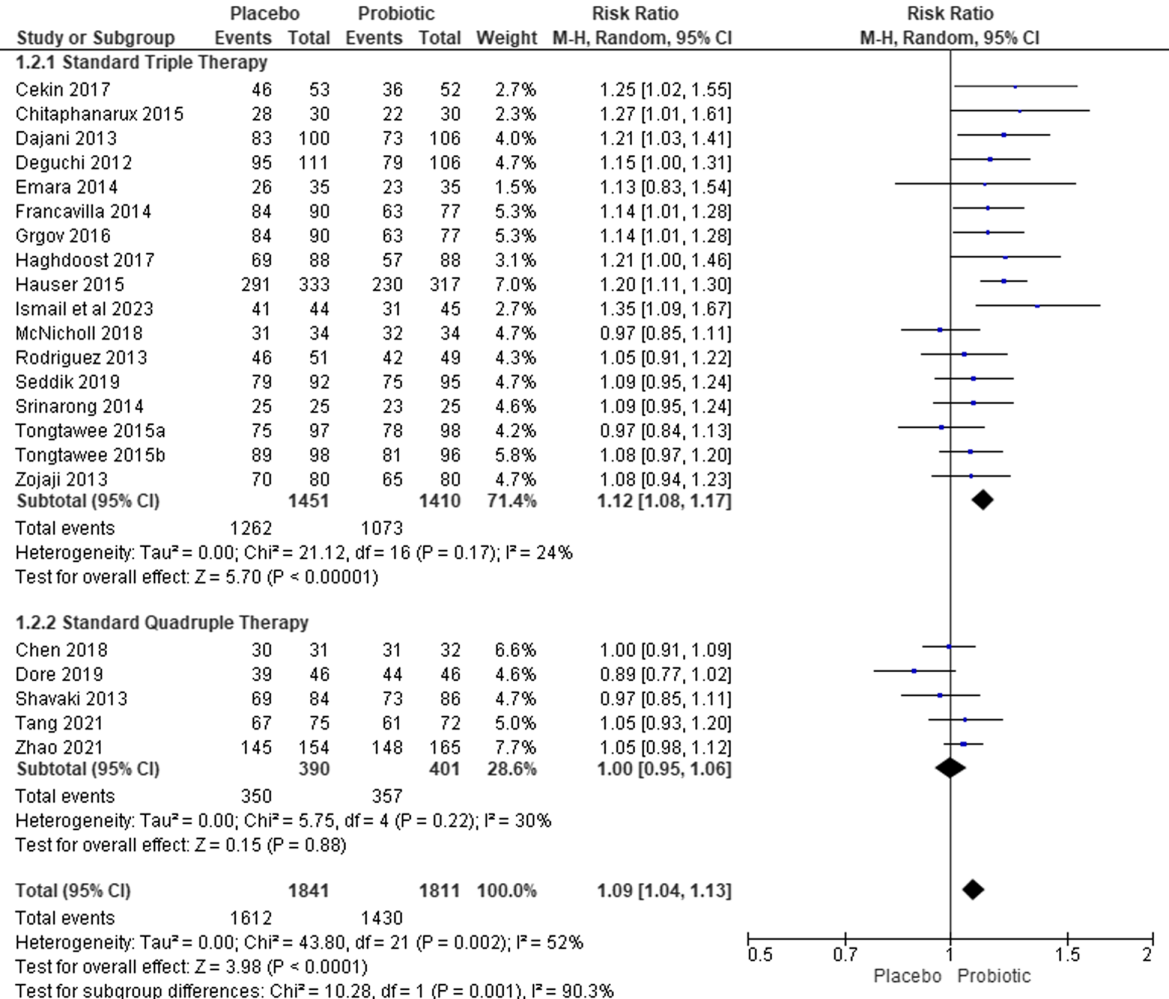
Meta-analysis for eradicating Helicobacter pylori with subgroup analysis based on the therapy regimen

**Fig. 3:**
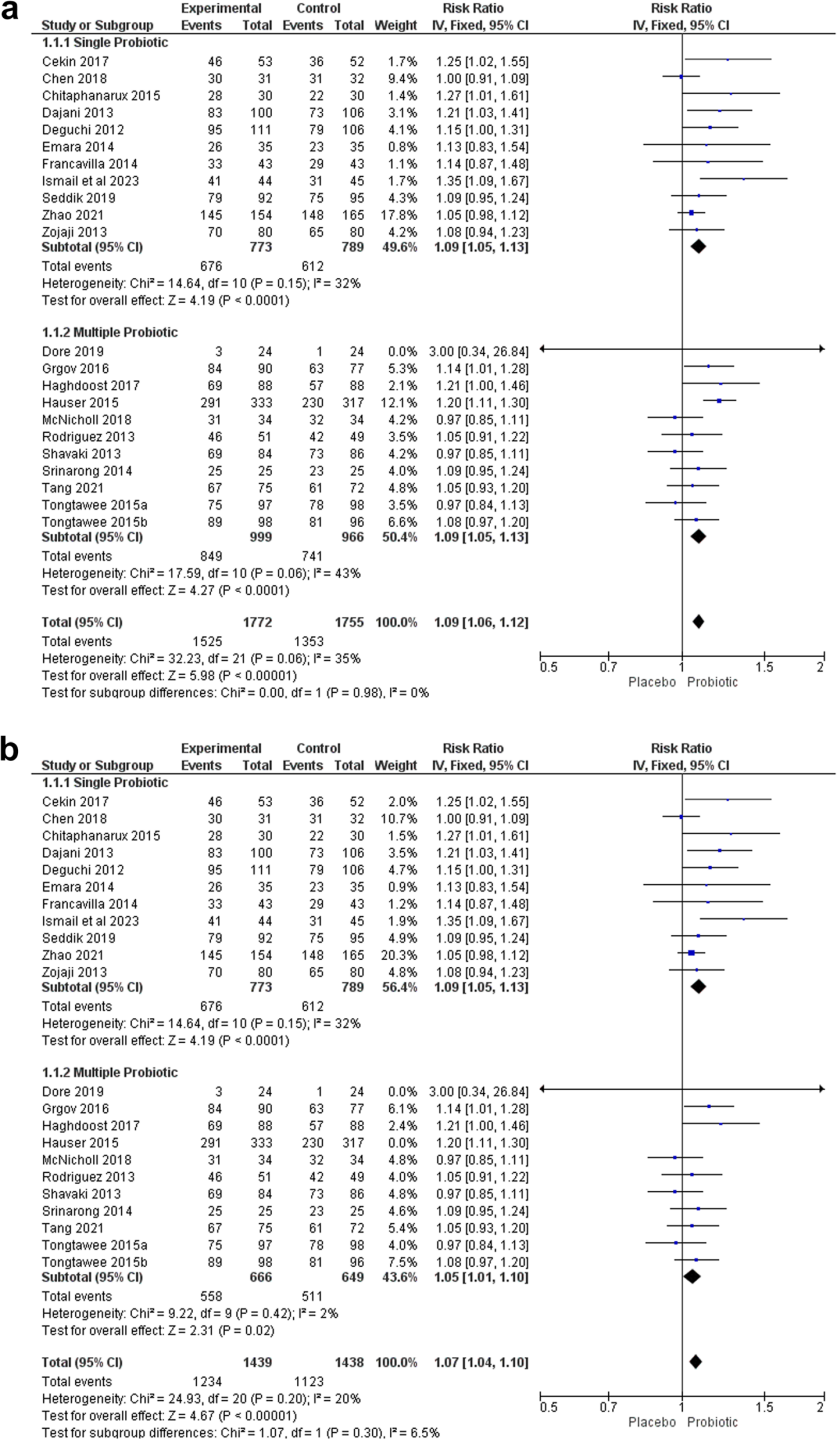
**a** Subgroup analysis based on the number of administered probiotics for eradicating Helicobacter pylori. **b** Sensitivity analysis by excluding studies by Hauser in the multiple probiotics subgroup

Overall, the included studies showed a low risk of bias and were relatively good studies. We found 22 studies that met the PICO criteria that involved 4,721 patients (Table 2). We divided the *H. pylori* analysis into two groups based on the standard therapy regimen (triple and quadruple) (Fig. 2) and the probiotic regimen (single or multiple strains) (Fig. 3a and b). Eighteen of twenty-two (82%) studies showed that regimen of probiotics is superior (RR ≥ 1.00) in achieving *H. pylori* eradication compared to the control group (Fig. 2). Nine out of twenty-two (41%) studies showed that regimen of probiotics could significantly eradicate *H. pylori* and was superior in achieving *H. pylori* eradication compared to the control group, however, the heterogeneity was high (RR 1.09, 95% CI 1.04–1.13, p value: 0.001, I^2^ = 52%) (Fig. 2).

#### Types of regimen subgroup analysis

Probiotics significantly improved *H pylori* eradication compared to placebo in the standard triple therapy group (RR 1.14, 95% CI 1.10–1.18, *p* value: < 0.001, I^2^ = 24%), but not in the quadruple therapy group (RR 1.01, 95% CI 0.96–1.06, p value: 0.62, I^2^ = 30%). Low heterogeneity was found in both standard triple and quadruple therapy or single and multiple probiotics. The funnel plot shows a symmetrical plot, which shows that studies included a low risk of bias (Supplementary Data 6).

#### Number of administered probiotics subgroup analysis

Subgroup analysis based on the number of administered probiotics showed that single probiotics had a same effect (RR 1.09 95% CI 1.05–1.13, *p* value: < 0.0001, I^2^ = 32%) than multiple probiotic regimens (RR 1.09 95% CI 1.05–1.13, *p* value: < 0.0001, I^2^ = 43%) as shown in Fig. 3a. Sensitivity analysis was performed by excluding the study by Hauser et al., which was performed in a younger population and involved more males than other studies, and resulted in a pooled RR of 1.07 (95% CI 1.04–1.10, *p*: < 0.0001), with low heterogeneity (I^2^ = 20%) (Fig. 3b).

#### Single probiotics subgroup analysis

**Fig. 4:**
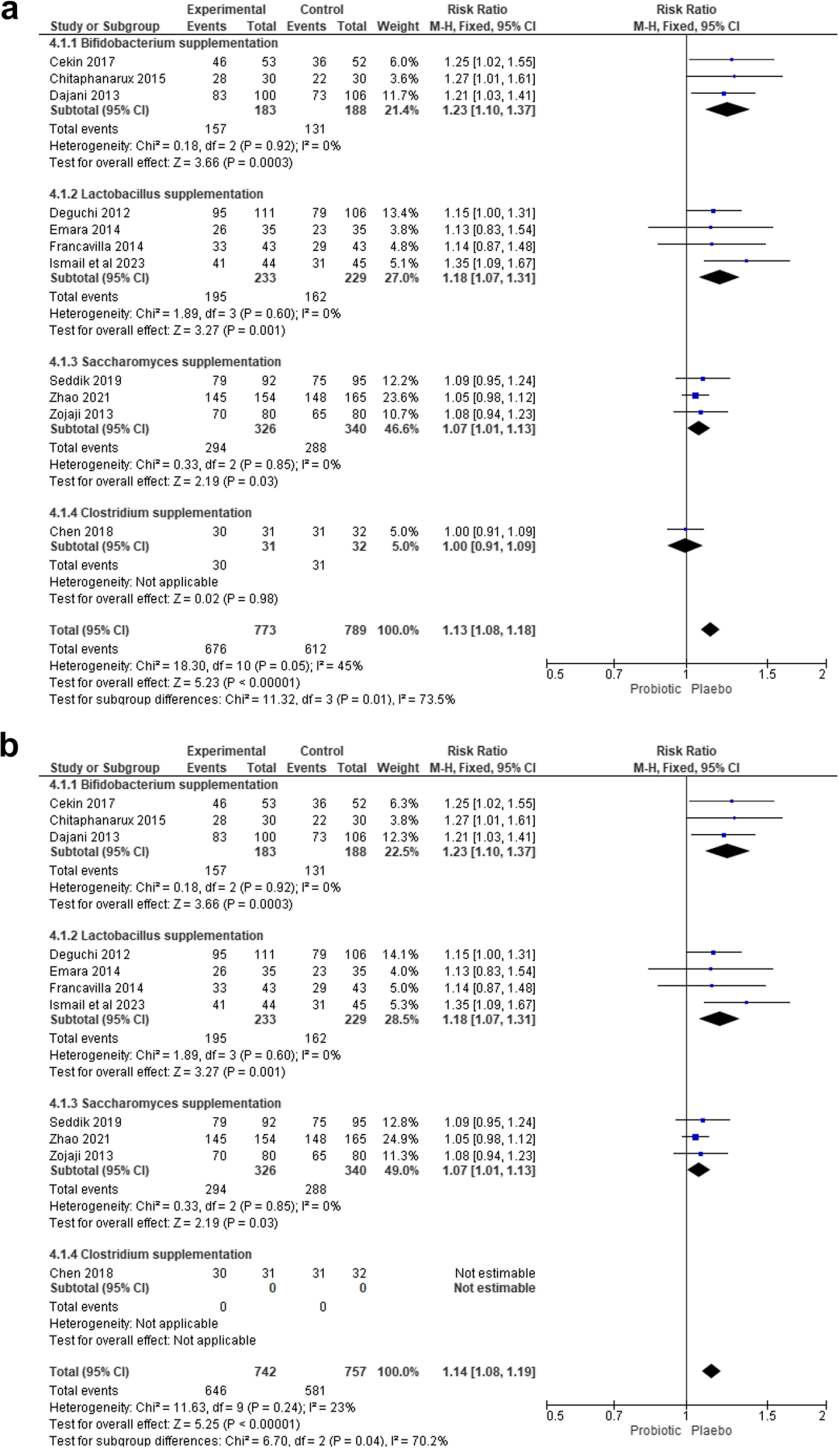
**a** Subgroup analysis based on the types of probiotics for eradicating Helicobacter pylori. **b** Sensitivity analysis by excluding studies by Chen in the Clostridium subgroup

Another subgroup analysis was performed by the type of probiotics used. We identified the single probiotic regimen used as members of the *Bifidobacterium, Lactobacillus, Saccharomyces*, and *Clostridium* families. The pooled RR for *Bifidobacterium* was 1.23 (95% CI 1.10–1.37, p value: 0.0003, I^2^ = 0%), for *Lactobacillus* 1.18 (95% CI 1.07–1.31, p: 0.001, I^2^ = 0%), and *Saccharomyces* 1.07 (95% CI 1.01–1.13, *p*: 0.03; I^2^ = 0%) (Fig. 4a). There was only one trial using *Clostridium* with an RR of 1.00 (95% CI 0,91–1.09, p: 0.98). Single probiotic regimen of *Bifidobacterium* appeared to have the highest curing success status.

Subgroup analysis was further performed to suggest which single probiotic has the highest efficacy. Our forest plot shows that groups that are given single *Bifidobacterium* probiotics produce the most superior effects compared to other single probiotics significantly, followed by *Lactobacillus*, *Saccharomyces*, and *Clostridium* single probiotics (1.23 vs 1.18; 1.07; 1.00) (Fig. 4a). Sensitivity analysis was then performed due to heterogeneity (I^2^ = 45%) (Fig. 4a), by excluding the study by Chen et al., which is the only *Clostridium* studies, and resulted in a pooled RR of 1.14 (95% CI 1.08–1.19, *p*: < 0.0001), with low heterogeneity (I^2^ = 33%) (Fig. 4b).

### *Probiotic and* UTI

**Fig. 5:**
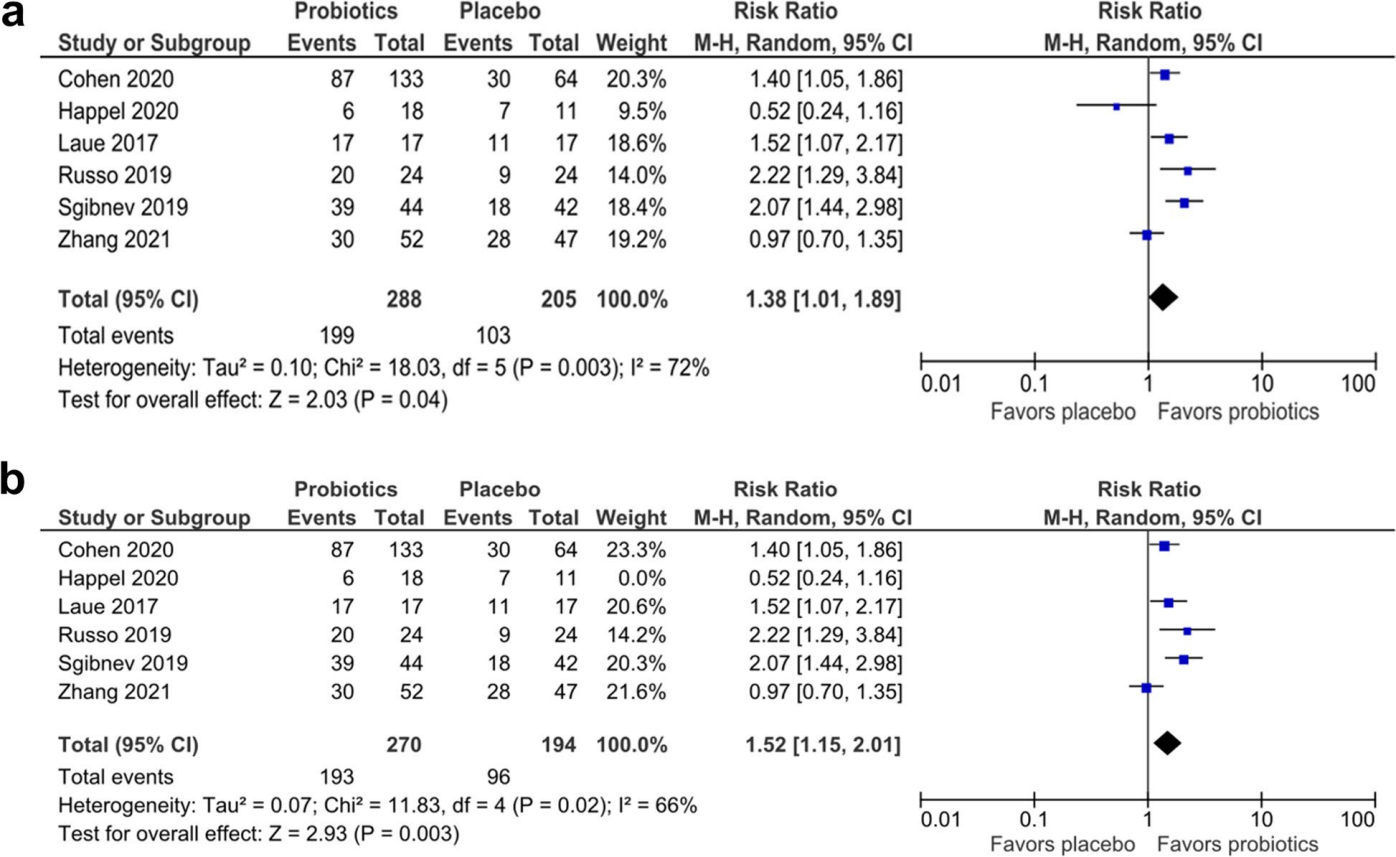
**a** Probiotics compared to placebo in achieving a cure range of Nugent score (< = 3) in UTI. **b** Sensitivity analysis of probiotics compared to placebo in achieving a cure range of Nugent score (< = 3) in UTI by excluding the study by Happel et al.

Our forest plot shows that the groups that were given probiotics had a better cure range (Nugent score ≤ 3) than the placebo group (RR 1.38: 95% CI 1.01–1.89, p: 0.04), although the heterogeneity was high (I^2^ = 72%), as shown in Fig. 5a. This has shown the potential of probiotics as a treatment for UTIs.

A sensitivity analysis by excluding the study by Happel [23], which was the only study that took place in Africa whereas others in America and European continents. It resulted in a higher pooled estimate with slightly lower heterogeneity (RR 1.52,95% CI 1.15–2.011.16, *p*: 0. 003; I^2^ = 66%) (Fig. 5b).

## Discussions

To our knowledge, this systematic review and meta-analysis is the first to summarize available evidence on the role of probiotics in treating common infectious diseases i.e., *H. pylori* infections, infectious diarrhea, urinary tract infections, and HIV infections.

### Probiotic and helicobacter pylori infection

In this study, the data generated from 23 heterogeneous studies demonstrated that the regimen of probiotics increased *H. pylori* eradication by 8% compared to the control group. Our findings suggest that probiotic supplementation might be used as an adjunctive therapy to improve the effectiveness of antibiotics. Several mechanisms are postulated to explain this finding. In a series of in vitro and in vivo studies, *L. reuteri* DSM 17648 has been shown to specifically bind to *H. pylori* in the gastric environment to form copolymers that interfere with its adhesion to the gastric mucosa and facilitate its elimination, thereby reducing the *H. pylori* load in the stomach [31, 35, 42]. Probiotics also aid in increasing the barrier effect of the stomach, which is the first line of defence against pathogenic bacteria [55]. Some probiotics can upregulate tight junction protein expression, promote mucin and mucus secretion and thus mucus secretion, and enhance the barrier effect of the gastric mucosa. Moreover, some probiotics can secrete antimicrobial substances, such as lactic acid, short-chain fatty acids (SCFAs), hydrogen peroxide, and bacteriocins. Organic acids can cause damage to *H. pylori* and inhibit its urease activity. Meanwhile, hydrogen peroxide and bacteriocins have direct antibacterial effects [26]. Probiotics are also able to interfere with the colonization of *H. pylori* in gastric mucosal epithelial cells by competing for adhesion sites, interfering with the adhesion process, and binding to *H. pylori* to form copolymers to facilitate its excretion ([30]). In terms of immune effects, probiotics may reduce the host inflammatory response by inhibiting the expression of proinflammatory factors [46]. We also conducted sensitivity analysis by excluding studies with the heaviest weights due to the high heterogeneity, which generated similar results (an 8% increase in the eradication rate).

Subgroup analysis based on therapy regimen showed that probiotics had better adjunctive effects in the standard triple therapy group than in the quadruple therapy group (10% vs 1% increase in the eradication rate). Importantly, our analysis also revealed that the increase in the eradication rate in quadruple therapy was not significant. This finding indicates that probiotic supplementation might offer less adjunctive effect in patients who have already been treated with quadruple therapy. The quadruple therapy is preferred as a first-line treatment in areas with a high incidence of clarithromycin resistance and as a second-line therapy after failure of the classical triple therapy. The finding in our analysis might be explained by the already higher cure rate with the use of quadruple therapy in several randomized controlled trials (RCTs) and a meta-analysis. In a multicenter RCT, the curing rate of bismuth quadruple therapy was significantly higher than that of standard triple therapy (90.4% vs 83.7%) for 14 days [36]. In a meta-analysis of Twenty-two randomized controlled trials (RCTs), diverse perspectives emerged. The eradication rate associated with triple therapy supplemented with probiotics exhibited a higher efficacy, in contrast to quadruple therapy, which did not demonstrate a uniform effect, aligning with the findings of our own studies ([63]). Notably, variations were observed in the geographical distribution of patients receiving quadruple therapy. As previously elucidated, quadruple therapy is recommended as the primary treatment in regions with elevated clarithromycin or metronidazole resistance. The meta-analysis encompassed diverse locations with varying resistance profiles, including those with high resistance, potentially influencing eradication rates. The consideration of various combinations of standard quadruple therapy in this meta-analysis further introduces potential variability in eradication rates across different locations. Despite the highly potent effects of H. pylori quadruple therapy, the addition of it may render its effects imperceptible. Consequently, the overall cure rates are anticipated to be influenced by participant demographics, the prevalence of susceptible infections, probiotics dosage and species, and the geographic variations in resistance patterns.

Another subgroup analysis compared single-strain probiotics to multi-strain probiotic regimens and showed that both had similar effects in increasing the eradication rate of *H. pylori*. Our finding is consistent with a previous systematic review and meta-analysis involving various types of infections. The study also demonstrated that the efficacy of multiple strains and single-strain probiotics were similar in their effectiveness [40]. The different efficacies of probiotic strains may be due to varying mechanisms of action possessed by different strains and if they are given singly or in combination with other strains. A clear advantage of a single strain has only been proven in necrotizing enterocolitis patients who receive *Lactobacillus rhamnosus* GG [43]. On the other hand, the efficacies of multi-strain probiotics might be enhanced if the mixture possesses synergistic effects, but vice versa if the effects are antagonistic. Eventually, the dynamic interactions between different strains in a mixture make the efficacies of multi-strain probiotics unpredictable. Therefore, the choice of an appropriate probiotic product for each specific disease will continue to be a clinical challenge and for cost-effectiveness, the decision must be based on available scientific evidence.

We also conducted a subgroup analysis comparing the single probiotic regimen by its families of bacteria (*Bifidobacterium, Lactobacillus, Saccharomyces*, and *Clostridium*). In our analysis, single probiotic regimen of *Bifidobacterium* appeared to deliver the highest increase in curing rate (23%). Bacteria belonging to the genus *Bifidobacterium* are among the first colonizers in the human gut after birth. Although the exact mechanism is not fully elucidated yet, numerous have been proposed mechanisms that account for this phenomenon, such as modulation of NFkB signaling and synthesis of antimicrobial peptides by *Bifidobacterium* [53]. Another important previous finding is the association between a low abundance of *Bifidobacterium* in the lower gut microbiota of *H. pylori*-infected patients [13]. Our findings support the use of *Bifidobacterium* as a probiotic supplement in *H. pylori* infection.

### Probiotic and urinary tract infection

Our analysis revealed that probiotics were superior (38% more decrease) in achieving a cure of UTI, indicated by a Nugent score of ≤ 3, compared to placebo as an adjunctive treatment to antibiotics. This effect might be accounted for by several mechanisms. Probiotics assist the work of antibiotics in treating UTI by binding to uroepithelial cells and inhibiting pathogenic growth and biosurfactant secretion. Oral *Lactobacillus* therapy can colonize these bacteria in the urinary tract following intestinal colonization [68]. The inhibition exerted by *Lactobacillus* sp. is mainly due to the release of lactic acid resulting from the metabolism of carbohydrates, which leads to a decrease in pH, making the environment hostile to most pathogens. The antagonistic activity of lactic acid seems to act synergistically with H_2_O_2_, which is also released by several *Lactobacillus* species in an aerobic environment [7]. The idea of oral probiotic application is based on the knowledge that pathogens that cause most urogenital infections progress from the rectum to the perineal region and then to the vagina and the mesentery [2]. In several studies, the antimicrobial activity of probiotics was tested by the agar diffusion method against reference strains or clinical isolates of urinary tract pathogens, mainly including enterobacteria, such as *E. coli*, *K. pneumoniae*, and *P. mirabilis*, and other bacteria, including *P. aeruginosa*, *E. faecalis*, and *S. saprophyticus* [52].

Sensitivity analysis was conducted by excluding the study with the lowest weight i.e., [23]. The result did not appear to favour probiotics to be utilized as an adjunctive therapy in the treatment of UTI. This indicated that the primary analysis result was greatly influenced by this one study due to the small number of participants. With this finding, future multi-center RCTs with a considerable number of participants are needed to confirm the effectiveness of probiotic supplementation as an adjunctive therapy for UTIs.

### Probiotic and infectious diarrhea

A meta-analysis was not performed for infectious diarrhea because there were only two eligible studies with different probiotic supplementations and outcome parameters. Nonetheless, they showed that diarrheal incidence was lower after regimen of a probiotic (*E. faecium* SF68) (T [20]) and complete remission of diarrheal was higher after the regimen of *B. coagulans* [37]. Probiotics used for diarrheal treatment mainly belong to the genera *Bacillus, Saccharomyces, Streptococcus, Lactobacillus, and Bifidobacterium*. The potential mechanisms by which probiotics fight infectious diarrhea include the exclusion of pathogens by means of competition for binding sites and available substrates, lowering of luminal pH and production of bacteriocins, and promotion of mucus production. Specific probiotic strains have been shown to normalize increased intestinal permeability and altered gut microecology, to promote intestinal barrier functions, and to alleviate the intestinal inflammatory response [29]. Further studies are needed to conduct a meta-analysis on the impact of probiotics on infectious diarrhea.

### Probiotic and HIV infection

A meta-analysis was not performed for HIV infection because the two eligible studies used different designs and comparators with contradicting findings. A crossover trial by Hemsworth et al. showed that CD4 declined after treatment with probiotics alone compared to micronutrients alone. In contrast, a two-arm RCT by Yang et al. yielded a higher percentage of blood CD4( +) T cells in the probiotics group than in the placebo group. HIV infection alters gut microbial ecology. HIV enteropathy includes pronounced gut-associated CD4^+^ T-cell loss and an impaired gastrointestinal (GI) epithelial barrier [45]. These detrimental changes presumably result in microbial translocation and a loss of gut homeostasis, which in turn leads to chronic immune activation and disease progression [19]. Hypothetically, probiotics oppose this effect by secreting polymeric IgA, avoiding the overgrowth and translocation of bacteria, and promoting the development of regulatory T cells through the production of anti-inflammatory cytokines [49]. Further studies are necessary to confirm the impact of probiotics on CD4^+^ cell count in HIV infection.

### Limitation

There are some limitations to our review. Except for the *H. pylori* study, our sample size was rather small for a meta-analysis of a few studies. We could not proceed with a meta-analysis for infectious diarrhea and HIV infection. The elevated heterogeneity observed in the study may be attributed to variations in data or design elements. These distinctions encompass differences in study target populations, targeted effects, methods of survey recruitment and administration, measurement instruments, intervention doses, timing of outcome measurements, analytical methods, and potential sources of bias, including adjustments for covariates [27]. Upon reviewing bias assessments utilizing both RoB 1 and RoB, it was observed that both assessments yielded similar conclusion regarding the presence of a high risk of bias. The primary distinction between these tools pertains to subjective outcomes in open-label studies, where RoB 1 tends to impose sanctions more frequently than RoB 2. Furthermore, RoB 1 is more prone to generating a heightened risk of biased judgments due to limited options, whereas RoB 2, with its integrated ratings, algorithms, signal questions, and guidance, facilitates a more straightforward assessment of complexity and context. Nonetheless, booth tools consistently showed that the majority of the studies had a low risk of bias. GRADE assessment indicated that the effect estimates of the use of single strain probiotics as adjuvant therapy in eradicating H. pylori and the use of probiotics in UTI had a high certainty of evidence. The effect estimates in other subgroups had a moderate certainty of evidence because some studies had a high risk of performance bias and/or conflicting interest with source of funding.

## Conclusion

In conclusion, this meta-analysis showed beneficial use of single strain probiotics as adjuvant therapy in eradicating H. pylori and the use of probiotics in UTI. Probiotic supplementation might not be beneficial for patients given a quadruple regimen. Single-strain and multi-strain probiotic regimens had similar effects in increasing the eradication rate of *H. pylori.* The benefits of probiotics as an additional regimen in infectious diarrhea and HIV infections remain unclear. Therefore more studies with more samples and effect sizes are still needed to confirm the benefits. Further studies are also needed to explore the potency of probiotics in another infection.

### Supplementary Information

**Supplementary Material 1.**

**Supplementary Material 2.**

**Supplementary Material 3.**

**Supplementary Material 4.**

**Supplementary Material 5.**

**Supplementary Material 6. **

**Supplementary Material 7. **

## Data Availability

The datasets generated and/or analysed during the current study are available in the Zenodo repository, https://zenodo.org/doi/10.5281/zenodo.10666345.

## Abbreviations

AAD: Antibiotic-Associated Diarrhea
AIDS: Acquired Immunodeficiency Syndrome
AH: Allerma Herdiman
ART: Antiretroviral Therapy
AGIK: Ayers Gilberth Ivano Kalaij
BV: Bacterial Vaginosis
BQT: Bismuth-containing Quadruple Therapy,
C: Control, CD4^+^: Cluster Differentiation 4
CFU: Colony-Forming Units
CI: Confidance Interval
DCs: Dendritic cells
ERA: Eradication
ESBL: Extended Spectrum**-**Β**-**Lactamase
FAO: Food Agriculture Organization
Fig: Figure
HAART: Highly Active Antiretroviral Therapy
HIV: Human Immunodeficiency Virus
*H. pylori*: *Helicobacter pylori*
HSV: Herpes Simpex Virus
ID: Infectious Diarrhea
IgA: Immunoglobulin A
I: Intervention
LAB: Lactic Acid Bacillus
LMIC: Low and Middle-Income Countries
MDs: Median Differences
MDR: Multidrug-Resistant
MSP2: Merozoite Surface Protein-2
NR: Not Reported
NK: Natural Killer
NS: Not significant (*p* value > 0.050)
*P*: *p* Value
PPI: Proton Pump Inhibitor
PRISMA: Preferred Reporting Items for Systematic Review and Meta-Analysis
PROSPERO: Prospective Register of Systematic Reviews
RCT: Randomized Controlled Trials
RKL: Richella Khansa Lauditta
RR: Risk Ratio
S: Significant (*p* value ≤ 0.050)
SMY: Syarif Maulana Yusuf
SQT: Standard Quadruple Therapy
STT: Standard Triple Therapy
TLR: Toll-Like Receptors
TV: *Trichomonas vaginalis*
UBT: Urea Breath Test
USA: United State of America
URTI: Upper Respiratory Tract Infection
UTI: Urinary Tract Infections
VRE: Vancomycin-Resistant *Enterococcus* spp.
WHO: World Health Organization
