# Supplementary material for: Role of probiotic as adjuvant in treating various infections: a systematic review and meta-analysis
Source: BMC Infect Dis. 2024 May 21;24:505. doi: 10.1186/s12879-024-09259-3 (PMC11106949; doi:10.1186/s12879-024-09259-3)
Supplement: Supplementary file 1 — Supplementary Material 1. [file 12879_2024_9259_MOESM1_ESM.docx]

**Supplementary Data 1. Source database and search strategy**

| Source Database | Text Availability | Document/ Source/ Publication Type | Article Type | Publication Date | Language |
| --- | --- | --- | --- | --- | --- |
| PubMed | Free full text | - | Clinical Trial, Randomized Controlled Trial | 2012 - 2024 | English |
| Scopus | All open access | Article/ Journal |  | 2012 - 2022 | English |
| Embase | - | Article | Clinical article, Clinical trial, Randomized controlled trial, Controlled clinical trial | 2012 - 2022 | - |
| Cochrane | - | - | - | 2012 - 2022 | - |

*Probiotics and H. pylori*

| **Database** | **Searching Strategy (Keyword)** | **Hits** |
| --- | --- | --- |
| PubMed (Jan 19^th^, 2024) | ("Probiotic"[Title/Abstract] OR "probiotics"[MeSH Terms]) AND ("H.pylori"[Title/Abstract] OR "helicobacter pylori"[Title/Abstract] OR "helicobacter pylori"[MeSH Terms] OR "helicobacter pylori"[MeSH Terms]) | 589 |
| Scopus (Jan 19^th^, 2024) | ( ( TITLE-ABS-KEY ( probiotic ) OR TITLE-ABS-KEY ( probiotics ) ) ) AND ( ( TITLE-ABS-KEY ( h.pylori ) OR TITLE-ABS-KEY ( helicobacter AND pylori ) ) ) | 1.625 |
| EMBASE (Jan 19^th^, 2024) | (“probiotics” and “*H. pylori*” or “*Helicobacter pylori*”) | 80 |
| Cochrane (Jan 19^th^, 2024) | ((("Probiotic"): ti,ab,kw OR (Probiotic):mesh)AND((Helicobacter Pylori):ti, ab,kw OR (Helicobacter Pylori): mesh))) | 203 |
| Total | | 2497 |

*Probiotics and Infectious-Diarrhea*

| **Database** | **Searching Strategy (Keyword)** | **Hits** |
| --- | --- | --- |
| PubMed (Jan 19^th^, 2024) | ("Probiotic"[Title/Abstract] OR "probiotics"[MeSH Terms]) AND ("infectious-diarrhea"[Title/Abstract] OR "infectious-diarrhea"[Title/Abstract] OR "dysentery"[MeSH Terms]) | 140 |
| Scopus (Jan 19^th^, 2024) | ( ( TITLE-ABS-KEY ( probiotic ) OR TITLE-ABS-KEY ( probiotics ) ) ) AND ( ( TITLE-ABS-KEY ( infectious-diarrhea ) OR TITLE-ABS-KEY ( infectious-diarrhea ) OR TITLE-ABS-KEY ( dysentery ) ) ) | 646 |
| EMBASE (Jan 19^th^, 2024) | (“probiotics” and “ID” or “Infectious-diarrhea”); (“probiotics” and “URTI” or “Upper Respiratory Tract Infection”) | 34 |
| Cochrane (Jan 19^th^, 2024) | ((("Probiotic"): ti,ab,kw OR (Probiotic):mesh)AND((infectious-diarrhea):ti, ab,kw OR (infectious-diarrhea): mesh))) | 179 |
| Total | | 999 |

*Probiotics and Upper Respiratory Tract Infection*

| **Database** | **Searching Strategy (Keyword)** | **Hits** |
| --- | --- | --- |
| PubMed (Jan 19^th^, 2024) | ("Probiotic"[Title/Abstract] OR "probiotics"[MeSH Terms]) AND ("URTI"[Title/Abstract] OR "upper respiratory tract infection"[Title/Abstract] OR "respiratory tract infections"[MeSH Terms]) | 667 |
| Scopus (Jan 19^th^, 2024) | ( ( TITLE-ABS-KEY ( probiotic ) OR TITLE-ABS-KEY ( probiotics ) ) ) AND ( ( TITLE-ABS-KEY ( urti ) OR TITLE-ABS-KEY ( upper AND respiratory AND tract AND infection ) ) ) | 432 |
| EMBASE (Jan 19^th^, 2024) | (“probiotics” and “URTI” or “Upper Respiratory Tract Infection”) | 76 |
| Cochrane (Jan 19^th^, 2024) | ((("Probiotic"): ti,ab,kw OR (Probiotic):mesh)AND((upper respiratory tract infection):ti, ab,kw OR (upper respiratory tract infection): mesh))) | 128 |
| Total | | 1303 |

*Probiotics and Urinary Tract Infection*

| **Database** | **Searching Strategy (Keyword)** | **Hits** |
| --- | --- | --- |
| PubMed (Jan 19^th^, 2024) | ("Probiotic"[Title/Abstract] OR "probiotics"[MeSH Terms]) AND ("UTI"[Title/Abstract] OR "urinary tract infection"[Title/Abstract] OR "urinary tract infections"[MeSH Terms]) | 223 |
| Scopus (Jan 19^th^, 2024) | (( TITLE-ABS-KEY ( probiotic ) OR TITLE-ABS-KEY ( probiotics ) ) ) AND ( ( TITLE-ABS-KEY ( uti ) OR TITLE-ABS-KEY ( urinary AND tract AND infection ) OR TITLE-ABS-KEY ( urinary AND tract AND infections ) ) ) | 865 |
| EMBASE (Jan 19^th^, 2024) | (“probiotics” and “UTI” or “Urinary Tract Infection”) | 58 |
| Cochrane (Jan 19^th^, 2024) | ((("Probiotic"): ti,ab,kw OR (Probiotic):mesh)AND((urinary tract infections):ti, ab,kw OR (urinary tract infections): mesh))) | 130 |
| Total | | 1276 |

*Probiotics and Human Immunodeficiency Virus*

| **Database** | **Searching Strategy (Keyword)** | **Hits** |
| --- | --- | --- |
| PubMed (Jan 19^th^, 2024) | ("Probiotic"[Title/Abstract] OR "probiotics"[MeSH Terms]) AND ("HIV"[Title/Abstract] OR "human immunodeficiency virus"[Title/Abstract] OR "HIV"[MeSH Terms]) | 199 |
| Scopus (Jan 19^th^, 2024) | ((TITLE-ABS-KEY ( probiotic) OR TITLE-ABS-KEY ( probiotics ))) AND ( ( TITLE-ABS-KEY ( hiv ) OR TITLE-ABS-KEY ( human AND immunodeficiency AND virus ))) | 582 |
| EMBASE (Jan 19^th^, 2024) | (“probiotics” and “HIV” or “Human Immunodeficiency Virus”) | 52 |
| Cochrane (Jan 19^th^, 2024) | ((("Probiotic"): ti,ab,kw OR (Probiotic):mesh)AND((human immunodeficiency virus):ti, ab,kw OR (human immunodeficiency virus): mesh))) | 42 |
| Total | | 875 |
