# Supplementary material for: Role of probiotic as adjuvant in treating various infections: a systematic review and meta-analysis
Source: BMC Infect Dis. 2024 May 21;24:505. doi: 10.1186/s12879-024-09259-3 (PMC11106949; doi:10.1186/s12879-024-09259-3)
Supplement: Supplementary file 2 — Supplementary Material 2. [file 12879_2024_9259_MOESM2_ESM.docx]

**Supplementary Data 2. RoB 1.0 and RoB 2.0 Risk of Bias Assessment**

**Table A. RoB 1.0 Tool risk of Bias**


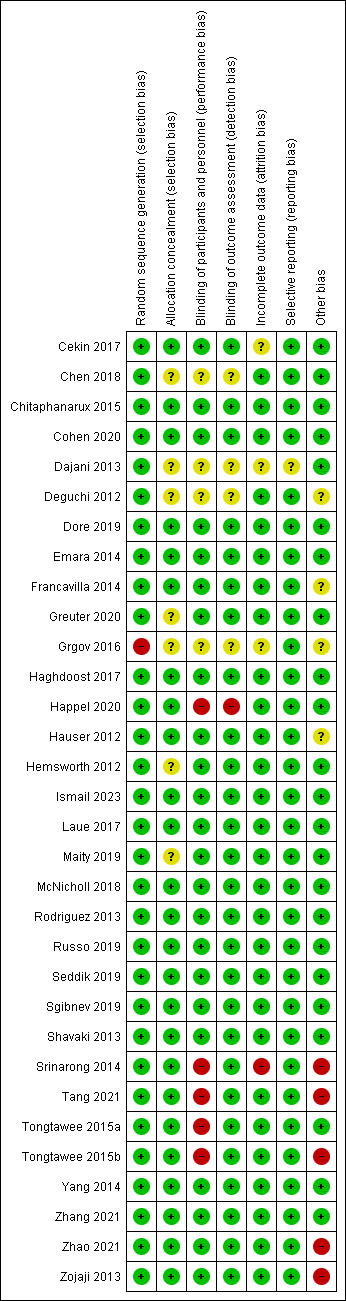

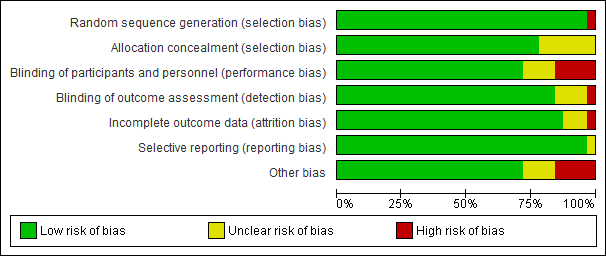


**Table B. RoB 2.0 Tool risk of Bias**

**
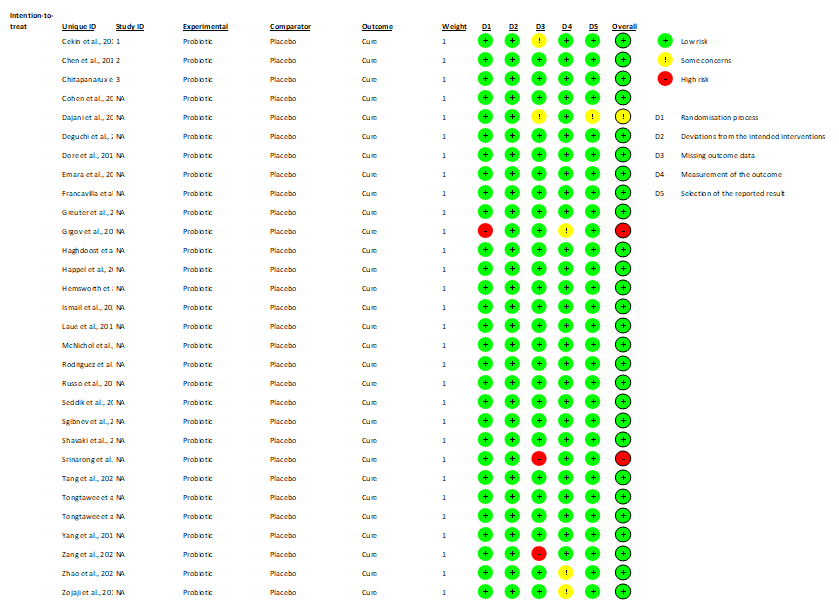
**
