# Supplementary material for: Role of probiotic as adjuvant in treating various infections: a systematic review and meta-analysis
Source: BMC Infect Dis. 2024 May 21;24:505. doi: 10.1186/s12879-024-09259-3 (PMC11106949; doi:10.1186/s12879-024-09259-3)
Supplement: Supplementary file 3 — Supplementary Material 3. [file 12879_2024_9259_MOESM3_ESM.docx]

**Supplementary Data 3: Table of GRADE Assessment Tool for Quality Appraisal.**

| **№ of studies** | **Certainty assessment** | | | | | | **Effect** | | | **Certainty** | **Importance** |
| --- | --- | --- | --- | --- | --- | --- | --- | --- | --- | --- | --- |
|  | **Study design** | **Risk of bias** | **Inconsistency** | **Indirectness** | **Imprecision** | **Other considerations (Associations)** | **№ of events** | **№ of individuals** | **Rate (95% CI)** |  |  |
| H. pylori cure (eradication rates – standard triple therapy) (follow-up: range 4 weeks to 8 weeks; assessed with: Risk ratio) | | | | | | | | | | | |
| 17 | Randomised controlled trials | Serious  3/17 studies had a high risk of performance bias, 4/17 studies had conflicting interest with source of funding. | not serious | not serious | not serious | Small important effect | 1073 | 1410 | RR 1.12 (95% CI: 1.08-1.17) | ⊕⊕⊕O Moderate | CRITICAL |
| H. pylori cure (eradication rates – standard quadruple therapy) (follow-up: range 4 weeks to 8 weeks; assessed with: Risk ratio) | | | | | | | | | | | |
| 5 | Randomised controlled trials | Serious  1/5 studies had a high risk of performance bias, 2/5 studies had conflicting interest with source of funding. | not serious | not serious | not serious | No effect | 357 | 401 | RR 1.00 (95% CI: 0.95-1.06) | ⊕⊕⊕O Moderate | CRITICAL |
| H. pylori cure (eradication rates – single probiotic) (follow-up: range 4 weeks to 8 weeks; assessed with: Risk ratio) | | | | | | | | | | | |
| 11 | Randomised controlled trials | not serious  no studies had a high risk of performance bias, 2/11 studies had conflicting interest with source of funding | not serious | not serious | not serious | Small important effect | 612 | 789 | RR 1.09 (95% CI: 1.05-1.13) | ⨁⨁⨁⨁ High | CRITICAL |
| H. pylori cure (eradication rates – multiple probiotics) (follow-up: range 4 weeks to 8 weeks; assessed with: Risk ratio) | | | | | | | | | | | |
| 11 | Randomised controlled trials | Serious  4/11 studies had a high risk of performance bias, 4/11 studies had conflicting interest with source of funding. | not serious | not serious | not serious | Small important effect | 741 | 966 | RR 1.09 (95% CI: 1.05-1.13) | ⊕⊕⊕O Moderate | CRITICAL |
| Urinary Tract Infections cure (Nugent Score) (follow-up: range 15 days to 24 weeks; assessed with: Risk ratio) | | | | | | | | | | | |
| 6 | Randomised controlled trials | not serious | not serious | not serious | not serious | Moderate effect | 103 | 205 | RR 1.36 (95% CI: 1.01-1.89) | ⨁⨁⨁⨁ High | CRITICAL |
