# Supplementary material for: Role of probiotic as adjuvant in treating various infections: a systematic review and meta-analysis
Source: BMC Infect Dis. 2024 May 21;24:505. doi: 10.1186/s12879-024-09259-3 (PMC11106949; doi:10.1186/s12879-024-09259-3)
Supplement: Supplementary file 5 — Supplementary Material 5. [file 12879_2024_9259_MOESM5_ESM.docx]

**Supplementary Data 5. Table of List Rejected Articles**

| Reasons reports excluded | Disease | Author and Publication Year | Title and DOI |
| --- | --- | --- | --- |
| Irrelevant clinical data (25 ) | Infectious Diarrheal | Lau et al., 2020 | Economic evaluation alongside the Probiotics to Prevent Severe Pneumonia and Endotracheal Colonization Trial (PROSPECT): study protocol (https://[10.1136/bmjopen-2019-036047](https://dx.doi.org/10.1136/bmjopen-2019-036047)) |
|  |  | Porcell et al., 2020 | Two vs. three weeks of treatment with amoxicillin-clavulanate for stabilized community-acquired complicated parapneumonic effusions. A preliminary non-inferiority, double-blind, randomized, controlled trial (https://[10.1515/pp-2019-0027](https://dx.doi.org/10.1515/pp-2019-0027)) |
|  |  | Warny et al., 2005 | Toxin production by an emerging strain of Clostridium difficile associated with outbreaks of severe disease in North America and Europe (https://[10.1016/S0140-6736(05)67420-X](https://doi.org/10.1016/s0140-6736(05)67420-x)) |
|  |  | Whittemore et al., 2018 | Short and long-term effects of a synbiotic on clinical signs, the fecal microbiome, and metabolomic profiles in healthy research cats receiving clindamycin: a randomized, controlled trial (https://[10.7717/peerj.5130](https://dx.doi.org/10.7717/peerj.5130)) |
|  | *H pylori* infections | Amal KM., 2023 | Effects of probiotics in adults with gastroenteritis: a systematic review and meta-analysis of clinical trial (<https://10.3390/disease11040138>) |
|  |  | Bai et al., 2023 | The impact of probiotics on gut microbiota in the eradication of *Helicobacter pylori* infections: a systematic review (<https://10.26355.eurrev_202307_33144>) |
|  |  | Baryshnikova et al., | Probiotics and autoprobiotics for treatment of *Helicobacter pylori* infection (<https://10.12998/wjcc.v11.i20.4740>) |
|  |  | Chen et al., 2019 | Probiotic Lactobacillus spp. Act Against Helicobacter pylori-induced Inflammation (https://[10.3390/jcm8010090](https://dx.doi.org/10.3390/jcm8010090)) |
|  |  | Dharshini et al., 2021 | Identification and Probiotic characterization of microbial communities from Fermented rice water and association of Enterococcus hirae with peptic ulcer causing Helicobacter pylori  (https://[10.22270/jmpas.V10I5.1436](https://dx.doi.org/10.22270/jmpas.V10I5.1436)) |
|  |  | Do et al., 2021 | Lactobacillus rhamnosus JB3 inhibits Helicobacter pylori infection through multiple molecular actions (https://[10.1111/hel.12806](https://dx.doi.org/10.1111/hel.12806)) |
|  |  | Gutierrez-Zamorano et al., 2019 | Increased anti-Helicobacter pylori effect of the probiotic Lactobacillus fermentum UCO-979C strain encapsulated in carrageenan evaluated in gastric simulations under fasting conditions (https://[10.1016/j.foodres.2018.12.064](https://dx.doi.org/10.1016/j.foodres.2018.12.064)) |
|  |  | Ji et al., 2018 | Efficacy of compound Lactobacillus acidophilus tablets combined with quadruple therapy for Helicobacter pylori eradication and its correlation with pH value in the stomach: a study protocol of a randomised, assessor-blinded, single-centre study |
|  |  | Ji et al., 2020 | Using Probiotics as Supplementation for Helicobacter pylori Antibiotic Therapy ([https://10.3390/ijms21031136](about:blank)) |
|  |  | Kakiuchi et al., 2020 | Effect of probiotics during vonoprazan-containing triple therapy on gut microbiota in Helicobacter pylori infection: A randomized controlled trial ([https://10.1111/hel.12690](about:blank)) |
|  |  | Kiecka et al., 2023 | Proton pump inhibitor-induced gut dysbiosis and immunomodulation: current knowledge and potentioal restoration by probiotics (<https://10.1007/s43440-023-00489-x>) |
|  |  | Kim et al., 2014 | Use of Selected Lactic Acid Bacteria in the Eradication of Helicobacter pylori Infection ([https://10.1007/s12275-014-4355-y](about:blank)) |
|  |  | Kouitcheu Mabeku  et al., 2020 | Potential of selected lactic acid bacteria from Theobroma cacao fermented fruit juice and cell-free supernatants from cultures as inhibitors of Helicobacter pylori and as good probiotic ([https://10.1186/s13104-020-4923-7](about:blank)) |
|  |  | Kumar et al., | Controlling intestinal infections and digestive disorders using probiotics (<https://10.1089/jmf.2023.0062>) |
|  |  | Lee et al., 2017 | Evaluation of the potential inhibitory activity of a combination of L. acidophilus, L. rhamnosus and L. sporogenes on Helicobacter pylori: A randomized double-blinxd placebo-controlled clinical trial ([https://10.1007/s11655-016-2531-0](about:blank)) |
|  |  | Musazadeh et al., 2023 | The effectiveness of treatment with probiotics in *Helicobacter pylory* eradication: results from an umbrella meta-analysis on meta-analyses of randomized controlled trials (<https://10.1039/d3fo00300k>) |
|  |  | Oh et al., 2016 | Changes in the Functional Potential of the Gut Microbiome Following Probiotic Supplementation during Helicobacter Pylori Treatment ([https://10.1111/hel.12306](about:blank)) |
|  |  | Park et al., 2021 | Microbiota changes with fermented kimchi contributed to either the amelioration or rejuvenation of helicobacter pylori-associated chronic atrophic gastritis ([https://10.3164/JCBN.20-123](about:blank)) |
|  |  | Peng., et al., 2023 | *Lactobacillus reuteri* in digestive system disease: focus on clinical trials and mechanisms (<https://10.3389/fcimb.2023.1254198>) |
|  |  | Wang et al., 2014 | Effect of Lactobacillus acidophilus and Bifidobacterium bifidum supplementation to standard triple therapy on Helicobacter pylori eradication and dynamic changes in intestinal flora ([https://10.1007/s11274-013-1490-2](about:blank)) |
|  |  | Wang et al., 2017 | Effects of anti-Helicobacter pylori concomitant therapy and probiotic supplementation on the throat and gut microbiota in humans ([https://10.1016/j.micpath.2017.05.035](about:blank)) |
|  |  | Wang et al., 2023 | Comparative effectiveness of different probiotics supplements for triple *Helicobacter pylori* eradication: a network meta-analysis (<https://10.3389/fcimb.2023.1120789>) |
|  |  | Yao et al., 2023 | Efficacy and safety of probiotic-supplemented bismuth quadluple therapy for the treatment of *Helicobacter pylori* infection: a systematic review and meta-analysis (<https://10.1177/03000605231203841>) |
|  |  | Yoon et al., 2019 | Fermented milk containing Lactobacillus paracasei and Glycyrrhiza glabra has a beneficial effect in patients with Helicobacter pylori infection: A randomized, double-blind, placebo-controlled study ([https://10.1097/MD.0000000000016601](about:blank)) |
|  | HIV Infections | Oyadiran et al., 2023 | Effect of intake of probiotics and probiotic fermented foods on clinical outcomes among people living with HIV: A systematic review and meta-analysis (https:// 10.1111/tmi.13955) |
|  |  | Stiksrud et al., 2015 | Reduced Levels of D-dimer and Changes in Gut Microbiota Composition After Probiotic Intervention in HIV-Infected Individuals on Stable ART ([https://10.1097/QAI.0000000000000784](about:blank)) |
|  | UTI | Bradshaw et ., 2012 | Efficacy of oral metronidazole with vaginal clindamycin or vaginal probiotic for bacterial vaginosis: Randomised placebo-controlled double-blind trial  ([https://10.1371/journal.pone.0034540](about:blank)) |
|  |  | Bustamante et al., 2020 | Probiotics and prebiotics potential for the care of skin, female urogenital tract, and respiratory tract  ([https://10.1007/s12223-019-00759-3](about:blank)) |
|  |  | El-Mekkawy et al., 2023 | Evaluation of the antibacterial activity of *Weissella confuse* K3 cell-free supernatant against extended-spectrum beta lactamase (ESBL) producing uropathogenic *Escherichia coli* U60 (<https://10.1016/j.sjbs.2023.103595>) |
|  |  | Laura et al., 20 | Biotics (Pre-, Pro-, Post-) and Uremic Toxicity: Implications, Mechanisms, and Possible Therapies (<https://10.3390/toxins15090548>) |
|  |  | Toh et al., 2019 | Probiotics [LGG-BB12 or RC14-GR1] versus placebo as prophylaxis for urinary tract infection in persons with spinal cord injury [ProSCIUTTU]: a randomised controlled trial  ([https://10.1038/s41393-019-0251-y](about:blank)) |
| Non-Placebo Studies (18) | *H pylori* infections | Di Pierro et al., 2020 | Impact of a two-bacterial-strain formula, containing Bifidobacterium animalis lactis BB-12 and Enterococcus faecium L3, administered before and after therapy for Helicobacter pylori eradication. (https://[10.23736/S1121-421X.19.02651-5](https://doi.org/10.23736/s1121-421x.19.02651-5)) |
|  |  | Dore et al., 2014 | Lactobacillus reuteri in the treatment of Helicobacter pylori infection ([https://10.1007/s11739-013-1013-z](about:blank)) |
|  |  | Jung et al., 2018 | Clinical Outcomes of Standard Triple Therapy Plus Probiotics or Concomitant Therapy for Helicobacter pylori Infection ([https://10.5009/gnl17177](about:blank)) |
|  |  | Khalil et al., 2015 | Efficacy of microencapsulated lactic acid bacteria in Helicobater pylori eradication therapy ([https://10.4103/1735-1995.172782](about:blank)) |
|  |  | Zhang et al., 2020 | The efficacy and safety of Clostridium butyricum and Bacillus coagulans in Helicobacter pylori eradication treatment: An open-label, single-arm pilot study.  ([https://10.1097/MD.0000000000022976](about:blank)) |
|  | HIV Infections | Hummelen et al., 2011 | Effect of Micronutrient and Probiotic Fortified Yogurt on Immune-Function of Anti-Retroviral Therapy Naive HIV Patients (https://[10.3390/nu310089](https://doi.org/10.3390/nu3100897)7) |
|  | UTI | Amstrong et al., 2022 | Sustained effect of LACTIN-V (Lactobacillus crispatus CTV-05) on genital immunology following standard bacterial vaginosis treatment: results from a randomised, placebo-controlled trial  (<https://10.1016/S2666-5247(22)00043-X>) |
|  |  | Lee et al., 2016 | Probiotics [LGG-BB12 or RC14-GR1] versus placebo as prophylaxis for urinary tract infection in persons with spinal cord injury [ProSCIUTTU]: a study protocol for a randomised controlled trial  (<https://10.1186/s12894-016-0136-8>) |
|  |  | Owen et al., 2019 | Protocol for a double-blind placebo-controlled trial to evaluate the efficacy of probiotics in reducing antibiotics for infection in care home residents: The Probiotics to Reduce Infections iN CarE home reSidentS (PRINCESS) trial (<https://10.1136/bmjopen-2018-027513>) |
|  | UTI | Carvalho et al., 2021 | Using lactobacilli to fight escherichia coli and staphylococcus aureus biofilms on urinary tract devices ([https://10.3390/antibiotics10121525](about:blank)) |
|  |  | Jiménez-Guerra  et al., 2017 | Urinary tract infection caused by Lactobacillus delbrueckii, a microorganism used as a probiotic  ([https://1058-4838/2004/3801-0009$15.00](about:blank)) |
|  |  | Ng et al., 2018 | Use of Lactobacillus spp. to prevent recurrent urinary tract infections in females  ([https://10.1016/j.mehy.2018.03.001](about:blank)) |
|  |  | Pathak et al., 2014 | Bifidobacterium friend or foe? A case of urinary tract infection with Bifidobacterium species  ([https://10.1136/bcr-2014-205122](about:blank)) |
|  |  | Shoureshi  et al., 2022 | Urinary bladder microbiome analysis and probiotic treatment options for women with recurrent urinary tract infections  ([https://](about:blank)10.1007/s00120-021-01621-6) |
|  |  | Tsai et al., 29018 | Evaluation of Lactic Acid Bacteria Isolated from Fermented Plant Products for Antagonistic Activity Against Urinary Tract Pathogen Staphylococcus saprophyticus  ([https://10.1007/s12602-017-9302-x](about:blank)) |
| Non-RCT studies (15) | Infectious Diarrheal | Hood et al., 2014 | Probiotics for Antibiotic-Associated Diarrhoea (PAAD): A prospective observational study of antibiotic-associated diarrhoea (including Clostridium difficile-associated diarrhoea) in care homes (https://[10.3310/hta18630](https://doi.org/10.3310/hta18630)) |
|  |  | Ouwehand et al., 2014 | Probiotics reduce symptoms of antibiotic use in a hospital setting: A randomized dose-response study (https://[10.1016/j.vaccine.2013.11.053](https://doi.org/10.1016/j.vaccine.2013.11.053)) |
|  | *H. pylori* infections | Dore et al., 2016 | Inclusion of Lactobacillus Reuteri in the Treatment of Helicobacter pylori in Sardinian Patients (https://[10.1097/MD.0000000000003411](https://dx.doi.org/10.1097/MD.0000000000003411)) |
|  |  | Drago et al., 2021 | Evaluation of main functional dyspepsia symptoms after probiotic administration in patients receiving conventional pharmacological therapies (https://[10.1177/0300060520982657](https://dx.doi.org/10.1177/0300060520982657)) |
|  |  | Efrati et al., 2012 | Helicobacter pylori eradication: Sequential therapy and Lactobacillus reuteri supplementation ([https://10.3748/wjg.v18.i43.6250](about:blank)) |
|  |  | Holz et al., 2015 | Significant Reduction in Helicobacter pylori Load in Humans with Non-viable Lactobacillus reuteri DSM17648: A Pilot Study ([https://10.1007/s12602-014-9181-3](about:blank)) |
|  |  | Ji et al., 2018 | Efficacy of compound Lactobacillus acidophilus tablets combined with quadruple therapy for Helicobacter pylori eradication and its correlation with pH value in the stomach: a study protocol of a randomised, assessorblinded, single-centre study (https://[10.1136/bmjopen-2018-023131](https://doi.org/10.1136/bmjopen-2018-023131)) |
|  |  | Klerk et al., 2016 | Lactobacilli ReduceHelicobacter pyloriAttachment to Host GastricEpithelial Cells by Inhibiting Adhesion Gene Expression (https://[10.1128/IAI.00163-16](https://dx.doi.org/10.1128/IAI.00163-16)) |
|  |  | Liu et al., 2020 | Treatment with compound Lactobacillus acidophilus followed by a tetracycline- and furazolidone-containing quadruple regimen as a rescue therapy for Helicobacter pylori infection  ([https://10.4103/sjg.SJG_589_19](about:blank)) |
|  |  | Mehling et al., 2013 | Non-viable lactobacillus reuteri DSMZ 17648 (Pylopass™) as a new approach to Helicobacter pylori control in humans ([https://10.3390/nu5083062](about:blank)) |
|  |  | Mukai et al., 2020 | Effectiveness of including probiotics to Helicobacter pylori eradication therapies  ([https://10.3164/jcbn.20-37](about:blank)) |
|  |  | Ohtsu et al., 2015 | The Ameliorating Effect of Lactobacillus gasseri OLL2716 on Functional Dyspepsia in Helicobacter pylori -Uninfected Individuals: A Randomized Controlled Study (https://[10.1159/000479000](https://doi.org/10.1159/000479000)) |
|  |  | Zagari et al., 202 | The “three-in-one” formulation of bismuth quadruple therapy for *Helicobacter pylori* eradication with or without probiotics supplementation: Efficacy and safety in daily clinical practice (<https://10.1111/hel.12502>) |
|  | HIV Infections | Falasca et al., 2015 | Effect of probiotic supplement on cytokine levels in HIV-infected individuals: A preliminary study (<https://doi.org/10.3390/nu7105396>) |
|  |  | Tenore et al., 2020 | Immune effects of *Lactobacillus casei* Shirota in treated HIV-infected patients with poor CD4+ T-cell recovery (https://[10.1097/QAD.0000000000002420](https://doi.org/10.1097/qad.0000000000002420)) |
|  |  | Villar et al., 2019 | Effects of Immunonutrition in Advanced Human Immunodeficiency Virus Disease: A Randomized Placebo-controlled Clinical Trial (Promaltia Study) (https://[10.1093/cid/ciy414](https://doi.org/10.1093/cid/ciy414)) |
|  | UTI | Bertuccioli et al., 2022 | Lactobacillus crispatus M247: Characteristics of a Precision Probiotic Instrument for Gynecological and Urinary Well-Being  ([https://10.3390/microbiolres13040069](about:blank)) |
| Irrelevant results (13) | Infectious Diarrheal | Beniwal et al., 2003 | A Randomized Trial of Yoghurt for Prevention of Antibiotic-associated Diarrhea (https://[10.1023/A:1026155328638](https://dx.doi.org/10.1023/A:1026155328638)) |
|  |  | Dietrich  et al., 2014 | Commercially available probiotic drinks containing Lactobacillus casei DN-114001 reduce antibiotic-associated diarrhea (https://[10.3748/wjg.v20.i42.15837](https://dx.doi.org/10.3748/wjg.v20.i42.15837)) |
|  |  | Ehrhardt et al., 2016 | Saccharomyces boulardii to Prevent Antibiotic-Associated Diarrhea: A Randomized, Double-Masked, Placebo-Controlled Trial (https://[10.1093/ofid/ofw011](https://dx.doi.org/10.1093/ofid/ofw011)) |
|  |  | Merenstein et al., 2009 | A Randomized Clinical Trial Measuring the Influence of Kefir on Antibiotic-Associated Diarrhea (https://[10.1001/archpediatrics.2009.119](https://dx.doi.org/10.1001/archpediatrics.2009.119)) |
|  |  | Psaradellis and Sampalis, 2010 | Efficacy of BIO K+ CL1285® in the reduction of antibioticassociated diarrhea – a placebo controlled double-blind randomized, multi-center study (https://[10.5114/aoms.2010.13508](https://dx.doi.org/10.5114/aoms.2010.13508)) |
|  |  | Rajkumar et al., 2020 | Do probiotics prevent antibiotic-associated diarrhoea? Results of a multicentre randomized placebo-controlled trial  (<https://doi.org/10.1016/j.jhin.2020.01.018>). |
|  |  | Safdar et al., 2008 | Feasibility and tolerability of probiotics for prevention of antibiotic-associated diarrhoea in hospitalised US military veterans (https://[10.1111/j.1365-2710.2008.00980.x](https://dx.doi.org/10.1111/j.1365-2710.2008.00980.x)) |
|  |  | Selinger et al., 2013 | Probiotic VSL#3 prevents antibiotic-associated diarrhoea in a double-blind, randomized, placebocontrolled clinical trial  (https://[10.1016/j.jhin.2013.02.019](https://dx.doi.org/10.1016/j.jhin.2013.02.019)) |
|  |  | Wong et al., 2014 | A Lactobacillus casei Shirota probiotic drink reduces antibiotic-associated diarrhoea in patients with spinal cord injuries: a randomised controlled trial (https://[10.1017/S0007114513002973](https://dx.doi.org/10.1017/S0007114513002973)) |
|  |  | Wong et al., 2021 | A study into the effect of Lactobacillus casei Shirota in preventing antibiotic associated diarrhoea including Clostridioides difficile infection in patients with spinal cord injuries: a multicentre randomised, doubleblind, placebo-controlled trial (https://[10.1016/j.eclinm.2021.101098](https://dx.doi.org/10.1016/j.eclinm.2021.101098)) |
|  | *H pylori* infection | Cárdenas et al., 2020 | Effect of Saccharomyces boulardii CNCM I-745 as complementary treatment of Helicobacter pylori infection on gut microbiome (https://[10.1007/s10096-020-03854-3](https://dx.doi.org/10.1007/s10096-020-03854-3)) |
|  |  | Cervantes-Elizarraras., 2019 | In vitro probiotic potential of lactic acid bacteria isolated from aguamiel and pulque and antibacterial activity against pathogens (https://[10.3390/app9030601](https://dx.doi.org/10.3390/app9030601)) |
|  |  | Fakhry SM., 2023 | Can probiotics play a role in *Helicobacter pylori* eradication? (<https://10.1186/s43066-023-00294-4>) |
|  | HIV infection | Blazquez-Bondia et al., 2022 | Probiotic effects on immunity and microbiome in HIV-1 discordant patients <https://10.3389/fimmu.2022.1066036> |
|  |  | Mohammadi-Sartang et al., 2018 | The effect of daily fortified yogurt consumption on weight loss in adults with metabolic syndrome: A 10-week randomized controlled trial (https://[10.1016/j.numecd.2018.03.001](https://dx.doi.org/10.1016/j.numecd.2018.03.001)) |
|  |  | Mortezazadeh et al., 2023 | The effect of oral probiotics on DC4 count in patients with HIV infection undergoing treatment with ART who have had an immunological failure (<https://10.1002/iid3.913>) |
|  | UTI | Gupta et al., 2023 | Effectiveness of phriphylactic oral and/or vaginal probiotic supplementation in the prevention of reccureent urinary tract infections: A randomized, double-blind, placebo-controlled trial (<https://10.1093/did/ciad766>) |
|  |  | New et al., 2022 | Role of probiotic for reccurent UTIs in the twenty-first century: a systematic review of literature (<https://10.1007/s1934-022-01085-x>) |
|  |  | Quattrone et al., 2023 | D-mannose plus *Saccharomyces boulardii* to prevent urinary tract infections and discomfort after cyctoscopy: a single-center prospective randomized pilot study (<https://10.3390/medicina59061165>) |
| Full texts were not available (8) | Infectious Diarrheal | Cimperman et al., 2011 | A Randomized, Double-blind, Placebo-controlled Pilot Study of *Lactobacillus reuteri* ATCC 55730 for the Prevention of Antibiotic-associated Diarrhea in Hospitalized Adults ([https://10.1097/MCG.0b013e3182166a42](about:blank)) |
|  |  | Li et al., 2010 | Use of probiotics for prevention of antibiotic-associated diarrhea in elderly patients  (https://[10.3969/j.issn.1008-7125.2010.03.007](https://dx.doi.org/10.3969/j.issn.1008-7125.2010.03.007)) |
|  |  | Pozzoni et al., 2012 | *Saccharomyces boulardii*for the Prevention of Antibiotic-Associated Diarrhea in Adult Hospitalized Patients: A Single-Center, Randomized, Double-Blind, Placebo-Controlled Trial ([https://10.1038/ajg.2012.56](about:blank)) |
|  |  | Velasco et al., 2019 | Probiotic Yogurt for the Prevention of Antibiotic-associated Diarrhea in Adults A Randomized Double-blind Placebo-controlled Trial (https://​​[10.1097/MCG.0000000000001131](https://dx.doi.org/10.1097/MCG.0000000000001131)) |
|  | *H. pylori* infection | Cifuentes et al., 2022 | *Saccharomyces boulardii* CNCM I-745 supplementation modifies the fecal resistome during *Helicobacter pylori* eradication therapy ([https://10.1111/hel.12870](about:blank)) |
|  |  | Devi et al., 2021 | Low Bifidobacterium Abundance in the Lower Gut Microbiota Is Associated with *Helicobacter pylori* -Related Gastric Ulcer and Gastric Cancer (https://[10.3389/fmicb.2021.631140](https://dx.doi.org/10.3389/fmicb.2021.631140)) |
|  |  | Marcial et al., 2017 | Exopolysaccharide-producing *Streptococcus thermophilus* CRL1190 reduces the inflammatory response caused by *Helicobacter pylori* (https://[10.3920/BM2016.0186](https://doi.org/10.3920/BM2016.0186)) |
|  |  | Miri et al., 2023 | Factors associated with treatment failure, and possible applications of probiotic bacteria in the arsenal against *Helicobacter pylori* (<https://10.1080/14787210.2023.2203382>) |
|  |  | Sjomina et al., 2023 | Randomised clinical trial: efficacy and safety of H. pylori eradication treatment with and without *Saccharomyces boulardii* supplementation (<https://10.1097/CEJ.0000000000000858>) |
|  | UTI | Chapman et al., 2014 | Effects of single- and multi-strain probiotics on biofilm formation and invitro adhesion to bladder cells by urinary tract pathogens  ([https://10.1016/j.anaerobe.2014.02.001](about:blank)) |
|  |  | Kyser et al., 2023 | Development and Characterization of Lactobacillus rhamnosus containing bioprints for application to catheter- associated urinary tract infection (<https://10.1021/acsbiomaterials.3c00210>) |
