# Supplementary material for: Role of probiotic as adjuvant in treating various infections: a systematic review and meta-analysis
Source: BMC Infect Dis. 2024 May 21;24:505. doi: 10.1186/s12879-024-09259-3 (PMC11106949; doi:10.1186/s12879-024-09259-3)
Supplement: Supplementary file 6 — Supplementary Material 6. [file 12879_2024_9259_MOESM6_ESM.docx]

**Supplementary Data 6. The funnel plot of meta-analysis for eradicating *Helicobacter pylori***


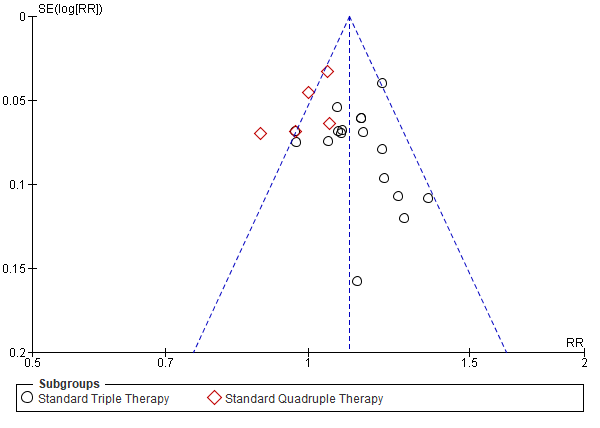


Fig 1. The funnel plot of meta-analysis for eradicating *Helicobacter pylori* with subgroup analysis based on the therapy regimen


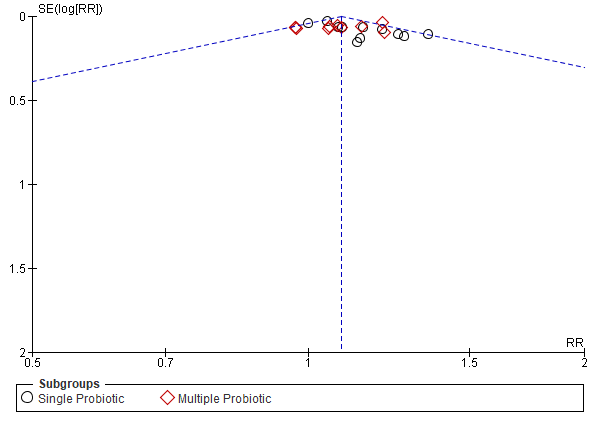


Fig 2. The funnel plot of meta-analysis for eradicating *Helicobacter pylori* with subgroup analysis based on the number of administered probiotics
